# The evolutionary origin of the Runx/CBFbeta transcription factors – Studies of the most basal metazoans

**DOI:** 10.1186/1471-2148-8-228

**Published:** 2008-08-05

**Authors:** James C Sullivan, Daniel Sher, Miriam Eisenstein, Katsuya Shigesada, Adam M Reitzel, Heather Marlow, Ditsa Levanon, Yoram Groner, John R Finnerty, Uri Gat

**Affiliations:** 1 Department of Biology, Boston University, 5 Cummington St. Boston, MA 02215, USA; 2 Department of Cell and Animal Biology, Silberman Institute of Life Sciences, Edmond Safra Campus at Givat-Ram, the Hebrew University, Jerusalem 91904, Israel; 3 Department of Chemical Research Support, the Weizmann Institute of Science, Rehovot 76100, Israel; 4 Faculty of Engineering, Doshisha University, Kyotanabe-Shi, 610-0394, Japan; 5 Department of Zoology, University of Hawai'i, Honolulu, HI 96822, USA; 6 Department of Molecular Genetics, the Weizmann Institute of Science, Rehovot 76100, Israel

## Abstract

**Background:**

Members of the Runx family of transcriptional regulators, which bind DNA as heterodimers with CBFβ, are known to play critical roles in embryonic development in many triploblastic animals such as mammals and insects. They are known to regulate basic developmental processes such as cell fate determination and cellular potency in multiple stem-cell types, including the sensory nerve cell progenitors of ganglia in mammals.

**Results:**

In this study, we detect and characterize the hitherto unexplored *Runx/CBFβ *genes of cnidarians and sponges, two basal animal lineages that are well known for their extensive regenerative capacity. Comparative structural modeling indicates that the Runx-CBFβ-DNA complex from most cnidarians and sponges is highly similar to that found in humans, with changes in the residues involved in Runx-CBFβ dimerization in either of the proteins mirrored by compensatory changes in the binding partner. *In situ *hybridization studies reveal that *Nematostella Runx *and *CBFβ *are expressed predominantly in small isolated foci at the base of the ectoderm of the tentacles in adult animals, possibly representing neurons or their progenitors.

**Conclusion:**

These results reveal that Runx and CBFβ likely functioned together to regulate transcription in the common ancestor of all metazoans, and the structure of the Runx-CBFβ-DNA complex has remained extremely conserved since the human-sponge divergence. The expression data suggest a hypothesis that these genes may have played a role in nerve cell differentiation or maintenance in the common ancestor of cnidarians and bilaterians.

## Background

Developmental processes occur throughout an animal's entire life history, during both pre-adult and adult stages. Just as patterning and morphogenesis are required to sculpt the body during embryogenesis and larval development, similar cellular differentiation pathways must be continuously re-deployed in the adult to compensate for the turnover of differentiated cells, to generate context-specific specialized cell types, and to repair damaged tissues. For example, in adult mammals, cellular differentiation pathways are activated to replace various cells of the hematopoietic lineage [1], to differentiate ova [2], to develop mature hair follicles [3], and to heal wounds [4]. Many such "adult developmental" processes are thought to depend upon stem cells exhibiting varying degrees of developmental potency [5]. Additionally, the same developmental processes may occur in those adult animals that are capable of extensively regenerating missing body parts [6].

One family of genes known to be involved in both pre-adult and adult development is the Runx family of transcription factors. Runx proteins are important for myriad developmental processes in both protostomes (*e.g.*, *Drosophila *and *Caenorhabditis*) and deuterostomes (*e.g.*, vertebrates and echinoderms) (reviewed in [7,8]). The Runx protein in a basal deuterostome, the sea urchin *Strongylocentrotus purpuratus *was found to be involved in basic developmental processes in the embryo and larva such as control of cell differentiation and survival [9-11], while an isoform of one of the zebrafish *Runx *genes products, Runx2b was recently found be a maternal factor acting as a ventral determining regulator in the earliest stages of axis formation in the embryo, further illustrating the importance of these factors in multiple aspects and phases of animal development [12]. Importantly, Runx factors have been also widely shown to play particularly key roles in cell fate determination and the maintenance of stem cell populations [13-15].

Some metazoans, including *C. elegans *and the sea urchin, have a single *Runt*-like gene, while others, including *Drosophila *and *Fugu*, may harbor up to four genes [7,8]. Mammalian genomes contain three *Runx *genes, each of which is critical for several different developmental processes occurring during embryogenesis: *Runx1 *is important for hematopoiesis and neurogenesis of distinct CNS neurons and nociceptive neurons in the dorsal root ganglion (DRG) [16-18], *Runx2 *is critical for skeletal morphogenesis [19-23], and *Runx3 *is important for neurogenesis of proprioceptive neurons in the DRG and for hematopoiesis [24,25]. Mammalian Runx proteins are also important for recurring developmental processes in the adult. For instance, loss of *Runx1 *in the adult causes hematopoietic abnormalities [26]. Overexpression of *Runx2 *in adults increases bone turnover leading to multiple fractures [27]. *Runx3 *controls CD8 cells, dendritic cells and CD4 Th1 cells development in adult stages [28].

*Runx *genes regulate transcription of target genes by binding to a variant of the consensus binding site RCCRCA (R = purine). Cis-regulatory Runx binding sites have been found in proximal promoter regions, as distant as a few thousand base pairs from the start methionine, in introns, and even within the coding region of genes whose transcription they regulate [29-31]. Runx binding is mediated by a highly conserved DNA-binding domain termed the Runt domain (RD) (reviewed in [8]). While the RD can bind DNA independently, *in vivo *binding usually occurs with its heterodimeric binding partner CBFβ. The protein CBFβ lacks a DNA-binding domain, but when bound to Runx, it increases the DNA-binding affinity of the RD several fold, substantially enhancing its transcriptional activity (reviewed in [32]). All presently known Runx proteins terminate in a short conserved pentapeptide motif, VWRPY, which binds the co-repressor Groucho and allows them to act as transcriptional repressors under certain conditions [33]. A post-translational control mechanism for mammalian Runx DNA-binding activity occurs via two cysteine 'redox switch' residues [34,35]. Interestingly, due to their ability to either activate or suppress target genes [36], when *Runx *genes are mutated or their regulation is perturbed, they can act as either tumor suppressors or tumor inducers.

**Figure 1:**
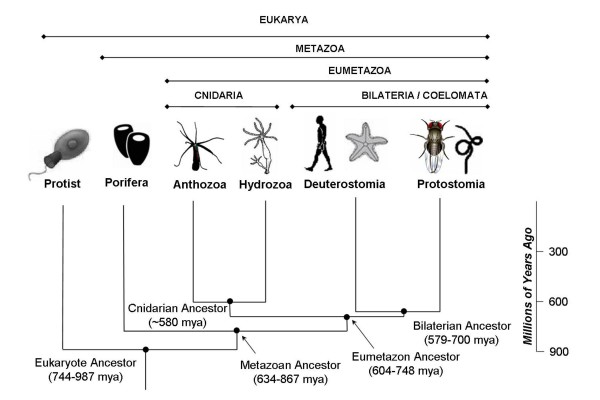
**Species level phylogeny for taxa utilized in this analysis**. Divergence time estimates are based on molecular clock estimates [80].

While *Runx *has been intensively studied in some triploblastic animals, little is known about its presence and function in the more ancient "basal" animal lineages such as cnidarians and sponges. These lineages are critical to understanding the early functional evolution of Runx because they diverged from the main metazoan stem lineage prior to the divergence of protostomes and deuterostomes (Figure 1). Over 99% of all extant animal species belong to the Triploblastica (or the Bilateria), including deuterostomes (*e.g.*, vertebrates and sea urchins) and protostomes (*e.g.*, insects and nematodes). Additionally, basal animals may be particularly informative about the evolution of developmental genes required during pre-adult and adult stages because they exhibit much greater regenerative capacity than many triploblastic model systems. For example, both sponges and cnidarians are able to completely regenerate from very small body fragments, and even from aggregates of single cells [37,38]. In cnidarians, this extensive regenerative capacity extends to the nervous system, which is able to regenerate in its entirety after injury. It is experimentally possible to eliminate the entire cnidarian nervous system, obtaining viable epithelial animals which regenerate a complete and functional nerve network when repopulated with interstitial stem cells [39,40]. During the regeneration process in hydra, pluripotent interstitial stem cells differentiate to form new nerve cells, gland cells and stinging neurons (nematocytes). In addition, the differentiated cells at the regenerating region undergo de-differentiation to form epithelial stem cells, which then re-differentiate to complete the regeneration process [41]. Sponges have only four cell types, and one of these cell types, the archaeocyte, is a stem-cell lineage that gives rise to the three other differentiated cell types [42,43]. Thus, cnidarians and sponges are excellent model organisms to study how developmental potency can be maintained in both stem cells and differentiated cells, and how de-differentiation and differentiation processes are initiated and controlled during *de-novo *creation of form upon regeneration.

In an effort to illuminate the early evolution of *Runx *and *CBFβ *genes and explore a possible ancestral connection with stem-cell potency and regeneration, we used computational methods to identify *Runx *and *CBFβ *orthologs from basal animals and then subjected them to phylogenetic analyses, comparative structural modeling, and expression studies. In the course of this work we identified *Runx *and *CBFβ *genes in cnidarians and sponges, revealing that these genes existed in the ancestral metazoan, an organism which lived perhaps as much as ~300 million years before the previously known earliest *Runx *and *CBFβ *genes from the bilaterian ancestor (Figure 1). The sequence of the Runt domain is highly conserved between sponges (*Amphimedon queenslandica *and *Oscarella carmela*), cnidarians (the sea anemone *Nematostella vectensis*, the freshwater hydra, *Hydra magnipapillata*, and the staghorn coral, *Acropora millepora*), and triploblastic animals. Likewise, the heterodimeric Runx/CBFβ transcription factor complex and its mode of DNA-binding appear to be highly conserved between sponges, cnidarians, and humans. *In situ *hybridization studies in adult *Nematostella *reveal expression in isolated cells of the tentacles, which may represent sensory neurons or their pluripotent progenitors. This possibility is intriguing because Runx factors are required for the development of sensory neurons in mammals [44-47]

## Results

### Novel *Runx *and *CBF*β genes in basal metazoans

To detect Runx and CBFβ genes in phylogenetically basal organisms, we used bioinformatic approaches to search EST and genomic databases for *Runx *and *CBFβ *in basal metazoans and unicellular protozoans. This search revealed well-conserved *Runx *and *CBFβ *gene pairs in the sea anemone *Nematostella vectensis*, the freshwater hydra *Hydra magnipapillata*, and the marine demosponge *Amphimedon queenslandica*. We also identified a *Runx *gene in the freshwater demosponge *Oscarella carmela *and a *CBFβ *gene in the coral *Acropora millepora*. Neither *Runx *nor *CBFβ *were detected in the genomes of any unicellular protozoans (Table 1).

**Table 1:** Accession numbers and genomic location of cnidarian and sponge *Runx *and *CBF*β genes.

	**Runx**			**CBF**β		
Taxon	Genbank	Genomic contig	Length(aa)	Genbank	Genomic contig	Length(aa)
		
*A. queenslandica*^†^	EU877200	gnl|ti|1463104027, gnl|ti|913734203	NA	EU877201	gnl|ti|858502066, gnl|ti|922473139	NA
O. carmela*	EC370682*	NA	NA	NA	NA	NA
*A. millepora*	NA	NA	NA	DY584722*	NA	NA
*H. magnipapillata*	EU877199	1101284938**	418	DT605894.1*	1101284935630** 1101284936757**	NA
*N. vectensis*	EU877198	c400502337***	496	EU877197	c418000979***	

† Predicted sequence

* EST Accession #

** Scaffold # for Venter Institute Draft Assembly

***Contig in Stellabase assembly v.1.0 [69].

The genomes of the following organisms were also searched, but no *Runx *genes were detected. (U) represents unfinished genomes at the time of the latest search (January 2008): Database: *Cryptosporidium hominis (U); Cryptosporidium parvum; Plasmodium berghei (U); Plasmodium chabaudi (U); Plasmodium falciparum 3D7; Plasmodium yoelii yoelii (U); Theileria parva (U); Theileria parva strain Muguga; Leishmania major strain Friedlin; Trypanosoma brucei TREU927; Trypanosoma cruzi (U); Dictyostelium discoideum AX4; Entamoeba histolytica HM-1:IMSS (U); Giardia lamblia ATCC 50803 (U)*.

### Runx

**Figure 2:**
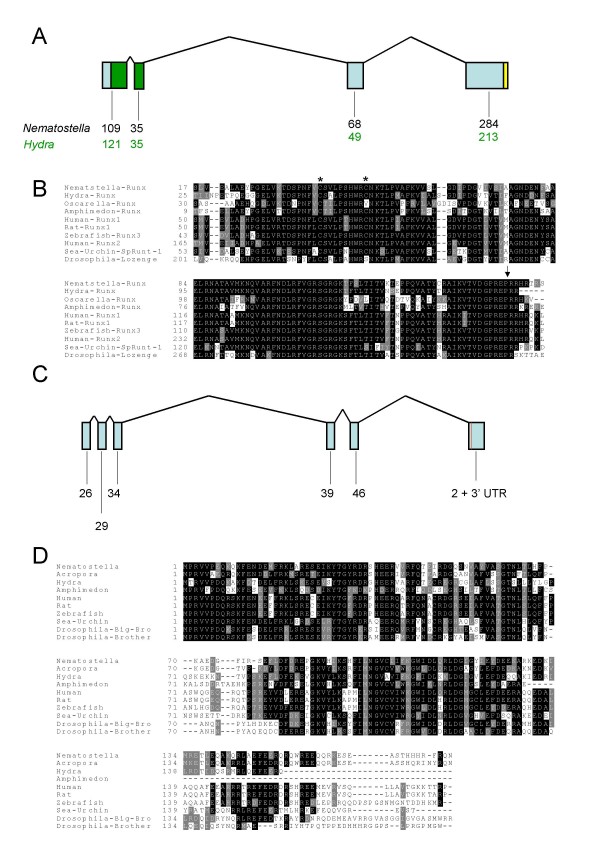
***Runx *and *CBFβ *genes in cnidarians and a sponge**. **A) Schematic representation of the genomic organization of the *Runx *gene from the sea-anemone *Nematostella vectensis***. Boxes indicate exons connected by lines representing introns. The Runt domain (RD) is in green. The conserved VWRPY Groucho-binding motif is in yellow. The numbers beneath the diagram represent the sizes of the various exon products (in amino acids) in the *Nematostella *and *Hydra *genes. **B) Conservation of the RD from sponges and cnidarians to mammals**. The arrowhead represents the end of the RD and * marks the cysteine residues which function as 'redox switches". **C) Schematic representation of the *CBFβ *gene from *Nematostella vectensis***. The red line in the last exon represents the location of the stop codon. The numbers beneath the diagram represent the sizes of the various exon products (in amino acids). **D) Conservation of CBFβ from cnidarians to mammals**.

The *Nematostella Runx *gene (*Nv-Runx*) encodes a predicted protein 496 amino acids long. It contains four exons, the first two of which encode the highly conserved Runt domain (RD). Within this domain, the *Nematostella *sequence is 81% identical and 93% similar to human RUNX1 (Figure 2A, B). The third and fourth exons encoding the C-terminus of the protein do not exhibit sequence similarity to other Runx proteins, nor to any other protein housed in the NCBI GenBank non-redundant database [48]. Nevertheless, as in other Runx proteins, the Nv-Runx C-terminal region, residues 148–495, is enriched in proline (13%), serine (9%) and threonine (8%) residues. The anemone protein ends with the canonical C-terminal VWRPY Groucho binding motif [33]. A characteristic intron at nucleotide position 328 within the RD, conserved amongst protostomes and deuterostomes [7], is also conserved in *Nv-Runx*. The second RD exon of *Nematostella *is identical in size to the corresponding exon in all deuterostome *Runx *genes (105 bp) that have been described. The closest similarity in gene structure is for the echinoderm Runx where the N-terminal part of the RD is encoded by a single exon while in other deuterostomes it spans two exons [7].

The *Hydra magnipapillata Runx *gene (*Hm-Runx*) encodes a shorter protein, 418 amino acids in length. The RD of Hm-Runx is also highly conserved with human Runx1 (82% identical and 92% similar). Hm-Runx also terminates in the Groucho binding motif VWRPY, but the remainder of this C-terminal region bears no obvious homology to any protein housed in GenBank. It is enriched in serine residues (14.8%), but interestingly, unlike the sea anemone protein, it is not enriched in proline (5.4%) or threonine (2.7%). The gene structure of *Hm-Runx *is similar to that of *Nv-Runx *and may thus reflect the ancestral condition for cnidarian *Runx *genes.

We found partial *Runx *transcripts from the freshwater demosponge *Oscarella carmela *(*Oc-Runx) *and the marine demosponge *Amphimedon queenslandica *(*Aq-Runx*) that encode well-conserved RDs. *Oc-Runx *and *Aq-Runx *share ~61% and 69% of residues with human RUNX1 in the 128 amino acid RD, respectively (Figure 2 and Additional file 1). The incomplete ESTs we identified do not encode the complete carboxy-terminus of the proteins, thus we cannot tell whether these sponge proteins also contain the Groucho-binding motif at their C-termini. However, all of the Runx proteins we have identified contain the two cysteine residues that act as redox switches in mammals (Figure 2, [34]). Notably, the second of these two cysteine residues (aligned residue 32 in Additional file 1) functions as a redox switch in deuterostomes but not in protostomes (being replaced by a serine residue in the latter). This position is a cysteine in both cnidarians and in one of the two sponge sequences (*A. queenslandica*), while being a valine in the freshwater sponge (*Oscarella carmela*).

### CBFβ

A search of the NCBI EST database revealed three cnidarian *CBFβ *ESTs – one from *Nematostella (Nv-CBFβ)*, one from *Hydra magnipapillata *(*Hm-CBFβ) *and one from a pre-settlement stage of the coral *Acropora millepora *(*Am-CBFβ*). The cnidarian ESTs encode proteins that are highly conserved relative to their bilaterian counterparts, *e.g.*, the *Nematostella *protein is 59% identical and 79% similar to the human CBFβ isoform 2 (Figure 2D, Additional file 2). The anemone and coral sequences are very similar to each other (74% identity and 93% similarity), whereas the *Hydra *protein is markedly more distant (58% identical and 80% similar to both the *Nematostella *and *A. millepora *variants). This reflects the ancient divergence of the medusozoan lineage, which includes *Hydra*, from the anthozoan lineage, which includes corals and sea anemones (Figure 1, [49,50]). A putative CBFβ protein identified in the genomic traces of the sponge *Amphimedon queenslandica *(*Aq-CBFβ) *bears almost as much similarity to human CBFβ as do cnidarian CBFβ proteins (54% identity, 73% similarity). Interestingly, *Aq-CBFβ *shares less identity with the 3 cnidarian CBFβ proteins (~47%) than it does with the human variant (54%). The sequence predicted based upon Blast homology includes exons from 2 genomic traces which cannot be directly assembled (Table 1).

The gene structure of *CBFβ *also appears to be highly conserved between cnidarians and deuterostome bilaterians. The *Nematostella CBFβ *(*Nv-CBFβ*) locus comprises six exons, and the positions of the first four exon boundaries are conserved relative to the *CBFβ *of the purple sea urchin, *Strongylocentrotus purpuratus *[11] (Figure 2C). The sixth exon is unique to *Nematostella *and encodes only the three final amino acid residues at the C-terminus of the protein, the stop codon, and the 3' UTR. The entire *CBFβ *transcript is encoded by a single exon gene in protostomes, suggesting that a retrotransposition event may have occurred deep within the protostome lineage.

### Cnidarian and Sponge Runx and CBFβ possess the structural elements needed for heterodimerization and DNA binding

In triploblastic animals, Runx and CBFβ proteins are known to cooperate in the regulation of gene expression by binding DNA as heterodimers (see [51] and references therein). To test whether the cnidarian and sponge variants of these proteins possess the structural determinants necessary for them to interact and bind DNA together in a manner homologous to the human complex, we modeled their three dimensional structure using the human AML1(RUNX1)RD-CBFβ-DNA complex as a modeling template (see Methods). Unless noted otherwise, all residue numbers described in the text below correspond to the human RUNX1 and CBFβ proteins (see Additional files 3, 4, 5 for a detailed comparison of the amino acids and their positions in the cnidarian and sponge proteins).

**Figure 3:**
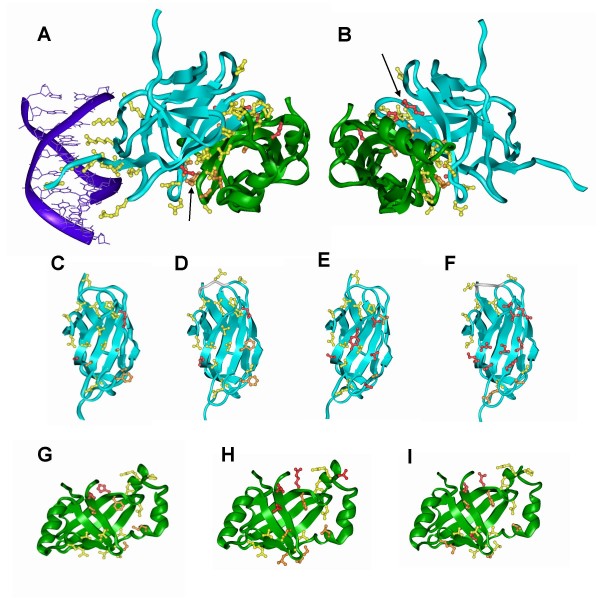
**Evolutionary conservation of the functional residues of Runx(RD) and CBFβ involved in DNA binding and dimerization**. The three dimensional structures of the cnidarian and sponge proteins were modeled according to the published structure of their human counterparts (PDB #1H9D). The RD is depicted in blue, CBFβ in green and DNA in purple. The interacting residues are depicted as ball and stick models, and colored according to their conservation with their human counterparts (Yellow = identical, Orange = conservative substitution, Red = non-conservative substitution). For clarity, a similar model with the relevant residues numbered according to the *Nematostella *Runx RD and CBFβ proteins is provided as Additional file 8, and detailed tables comparing the residues of all the proteins discussed can be found as Additional files 3, 4, 5. **A) A General view of the *Nematostella *RD-CBFβ-DNA complex**. All of the residues in the RD involved in DNA binding are completely conserved in *Nematostella*. The arrow points to compensatory changes in H163 of the RD, which is replaced with C131 in *Nematostella*. **B) A rotation of Panel A by 180°**, showing a second compensatory change which involves the substitution of F153 and M106 in human with K121 and A73 in *Nematostella *in the RD and the replacement of Q67 and S65 with H67 and T65 in CBFβ (indicated with arrow). **C-F) The CBFβ-binding faces of the RD from *Nematostella *(C), *Hydra *(D), *Amphimedon queenslandica *(E) and *Oscarella carmella *(F)**. In the cnidarian proteins, the majority of non-conserved residues which interact with CBFβ are located at the periphery of the interacting surfaces (C, D). *A. queenslandica *and particularly *O. carmella *indicate greater sequence divergence within this domain, with non-conservative substituions not being restricted to the periphery. **G-I) the Runx-binding surfaces of CBFβ proteins from *Nematostella *(G), *Hydra *(H) and *A. queenslandica *(I)**. Most of the functional residues are conserved between all three proteins and the human variant, with most of the non-conservative subsitutions found at the periphery of the binding face.

Figure 3A shows a general view of the model of the *Nematostella *RD-CBFβ dimer in complex with DNA, with the functional residues involved in RD-CBFβ dimerization and DNA binding highlighted as ball-and-stick models. The RD contains two distinct functional surfaces – one involved in DNA binding (composed of 10 functional residues) and one mediating dimerization with CBFβ. CBFβ itself does not directly interact with the DNA double helix. Strikingly, all 10 residues of the DNA binding face of all of the cnidarian and sponge RD's are completely conserved with their human counterpart, strongly indicating that these proteins bind DNA in a similar manner (Figure 3A, Additional file 3 and 6) and prefer the same DNA sequence motif.

In three of the four organisms – *Nematostella*, *Hydra *and *Amphimedon *– the dimerization faces of Runx and CBFβ are conserved, and when amino acid residues differ from those of the human proteins these differences do not alter the general structure of the protein complex (Figure 3C–E, G–I, Additional files 4, 5). This can best be demonstrated with a more detailed analysis of the *Nematostella *Runx-CBFβ-DNA complex: in Nv-Runx, 13 of the 19 residues on the CBFβ binding face are conserved, including all P and G residues that may be important for the folding of the polypeptide chain (Figure 3C, Additional file 4). Four additional residues (M106, Y113, S114 and T147) are replaced by amino acids with similar physical-chemical characteristics, and only two residues (F153 and H163) are significantly different. The latter two residues are found in the outer rim of the interface where protein-protein contacts are generally less conserved. Similarly, 12 of the 18 residues involved in Runx binding by the human CBFβ are identical in Nv-CBFβ. Four additional residues have conserved physical-chemical characteristics (F17, T60, S65 and M101), and 2 residues (Q67 and P100) are significantly different (Figure 3G, Additional file 5).

It is noteworthy that the latter residues, while different, are well accommodated within the structure of the proteins: F153 of human Runx, which is in a hydrophobic environment (L102, T104, T121, T151), is replaced by K in Nv-Runx, whose side chain is capable of making most of the interactions with the hydrophobic environment. The positively charged end of this K makes an H-bond with the S that replaces T104. H163 in human Runx1 makes an exposed interaction (and therefore of small energetic contribution) with D66; it is replaced by C in Nv-Runx, which cannot form such an interaction but can interact with the nearby backbone carbonyl. P100 of human CBFβ, which makes hydrophobic contacts with Y85 and I109, is replaced by H in Nv-CBFβ. This residue can form a hydrogen bond with the backbone carbonyl of F69. Q67 of human CBFβ is solvent exposed in the separated molecule: it is replaced by H in *Nematostella *without losing or gaining particular contacts.

Interestingly, while not all of the residues in the RD-CBFβ interface are conserved between *Nematostella *and humans, a change in the residue of one protein is accompanied by a reciprocal change in its binding partner (Figure 3B). One such change involves residues F153 and M106 in human Runx1 RD, which interface with S65 and Q67 of CBFβ. *Nematostella *differs at both positions in its RD, where F153 and M106 are replaced by K121 and A73 in the *Nematostella *protein; these changes in the Runx protein are mirrored by changes in CBFβ, where S65 and Q67 are replaced by T65 and H67 (Figure 3E). Another reciprocal change involves H163 of human Runx1 RD, which interfaces with T62 and F17 in CBFβ; the corresponding residues in *Nematostella *are C131 (in RD) and S62 and M17 (in CBFβ), respectively. This suggests that while the specific residues may differ between taxa, these proteins have co-evolved to maintain their functional interaction.

In contrast to the Runx proteins from the cnidarians and the demosponge *Amphimedon*, the CBFβ binding face of the sponge *Oscarella carmela *Runx protein is quite distinct, with many non-conservative amino acid substitutions found both in the periphery and in the center of the CBFβ binding region (Figure 3F). This suggests either that the corresponding CBFβ protein in this sponge is considerably different, or that the Oc-Runx does not bind a CBFβ protein at all.

### Phylogenetic history of Runx and CBFβ

*Runx *and *CBFβ *genes have previously been reported in many different phyla of triploblastic animals where a single species may contain multiple *Runx *or *CBFβ *genes. The detection of *Runx *and *CBFβ *genes in cnidarians and sponges allows, for the first time, reconstruction of their early evolutionary history, in the period preceding the evolutionary origin of Triploblasts.

**Figure 4:**
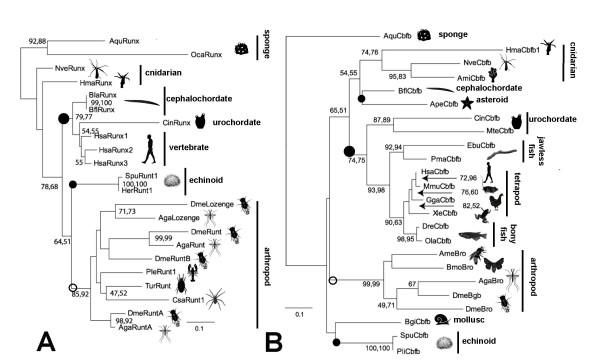
**Phylogeny of Runx (A) and CBFβ (B) proteins**. In both cases, maximum likelihood trees were constructed using the program PhyML. In the case of Runx, likelihoods were calculated using the Rt-REV amino acid substitution matrix [83]. In the case of CBFβ, likelihoods were calculated using the JTT amino acid substitution matrix. Separate neighbor-joining analyses using the JTT distance matrix were also performed for each data set (not shown). The bars and icons indicate the locations of the taxonomic groupings on each tree with the deuterostome and protostome groups marked with filled and empty circles respectively. Both trees are rooted between the sponge sequences and the eumetazoan sequences. The length of each horizontal branch is proportional to the number of amino acid substitutions that have occurred along that branch (scale at lower part of each panel). The first and second numbers at each node indicate the percentage of 1,000 bootstrap replicates in which the given clade is recovered with maximum likelihood and neighbor-joining analyses, respectively. Nodes which failed to receive at least 40% bootstrap support in one of the analyses are not labeled. The sources of the sequences and the abbreviations for taxa are provided in Additional files 9 and 10.

Figure 4A depicts a phylogeny of Runx proteins from sponges, cnidarians, chordates, echinoderms, and arthropods. Sequences from the nematodes *C. elegans*, *C. brigsae*, and *D. coronatus *were excluded from this analysis as they are highly divergent from the other metazoan Runx proteins, and their inclusion would tend to cause long-branch attraction artifacts. An analysis including the nematode species is presented in Additional file 6 (see Additional file 9 for taxon IDs). The tree is rooted using the two sponge sequences. Consistent with the prevailing view of organismal phylogeny, this gene tree groups the two sponge sequences, the eighteen triploblast sequences, the six chordate sequences, the two urchin sequences, and the ten arthropod sequences into mutually exclusive clades, with each clade receiving bootstrap support > 75%. The Runx sequences do not recover a monophyletic Deuterostomia as the two sea urchin sequences (*Strongylocentrotus *and *Heliocidaris*) group more closely with insects than with chordates (although the bootstrap support for this grouping is relatively low). Neither does the Runx tree support cnidarian monophyly, as the sea anemone and hydra sequences appear as successive outgroups at the base of the triploblast clade. Interestingly, this tree implies that the cnidarian proteins have evolved relatively slowly. In particular, the sea anemone Runx appears to have undergone the least amount of evolutionary change of any taxon since the time of the sponge-triploblast ancestor. The anemone may therefore represent our best source of information regarding the ancestral Runx protein. As the human Runx paralogs (Runx1–3) and the insect Runx paralogs (e.g., Drosophila Lozenge, Runt, RuntA, and RuntB) fall into distinct and well supported clades, this tree implies that the ancestral Runx gene underwent independent gene duplication in the deuterostome and protostome lineages.

Figure 4B depicts a phylogeny of CBFβ proteins that is rooted using the single sequence from the sponge *Amphimedon*. Once again, the nematode sequences were found to be highly divergent from other metazoan CBFβ proteins, and they were excluded from this analysis. A tree containing these sequences is presented as Additional file 7 (see Additional file 10 for taxon IDs). Like the Runx tree, this gene tree recovers the monophyly of a number of accepted taxonomic groups. It groups the three cnidarian sequences, the two sea urchin sequences, the five insect sequences, the two ascidian sequences and the eight vertebrate sequences. Furthermore, the vertebrate clade is subdivided among three well established monophyletic taxa: the two jawless fishes (*Eptatretus *and *Petromyzon*), the two ray-finned fishes (*Danio *and *Oryzias*), and the four tetrapods (*Xenopus, Gallus, Mus, and Homo*). Once again, as in the Runx tree, the sea urchin sequences (*Strongylocentrotus *and *Paracentrotus*) do not group with other deuterostome sequences or even with the other echinoderm sequence in the analysis, the sea star *Asterina*. The tree is not consistent with triploblast monophyly because the cnidarian sequences fall within a cluster of deuterostome sequences.

**Figure 5:**
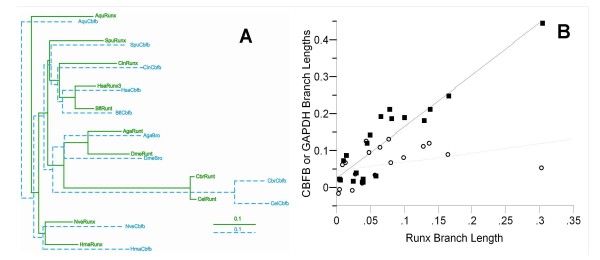
**Co-evolution of Runx and CBFβ proteins**. **A) **We generated the accepted organismal phylogeny relating 11 taxa for which both Runx and CBFβ sequences are available. We then inferred the evolutionary change in Runx (blue) and CBFβ (green) along each branch of this phylogeny by using the JTT matrix to calculate patristic distances. **B) **For each node on the tree, the evolutionary change in CBFβ and GAPDH were plotted against the evolutionary change in Runx. The regression reveals a significant and strong correlation between the evolutionary change in Runx and CBFβ (represented by squares and solid line; *R*^2 ^= 0.83, *F*_1,17 _= 85.11, *p *< 0.0001) but not between Runx and GAPDH (circles and dashed line). The outliers (the circle and square with a Runx branch length of ~0.3) represent the divergence between the protostome ancestor and the common *Caenorhabditis *ancestor (see panel A). Both Runx (branch length = ~0.3) and CBFβ (branch length = ~0.44) diverged rapidly within this lineage, while GAPDH continued to evolve more slowly (equivalent branch length in GAPDH tree = ~0.05).

In addition to the apparent reciprocity of amino acid changes that we have observed in the interacting portions of Runx and CBFβ, the anomalous position of the sea urchin sequences on both the Runx and CBFβ trees could be another reflection of co-evolution between these proteins. To explore this hypothesis further, we employed a variation on the protein co-evolution test proposed by Sato and coworkers [52] (Figure 5). When each of the phylogenies described above is limited to only those 11 taxa contained in both phylogenies, and the tree topologies are constrained to the accepted organismal phylogeny (Figure. 5A), the resultant branch lengths on the two trees are significantly and strongly correlated (Figure 5B, *R*^2 ^= 0.83; Linear Least Regression test for significance: *F*_1,17 _= 85.11, *p *< 0.0001). In contrast, the branch lengths from a glyceraldehyde-phosphate-dehydrogenase phylogeny, which is a "house-keeping" gene serving as a control, are not correlated with the Runx branch lengths (*F*_1,17 _= 2.73, *p *= 0.1167) and only weakly correlated with the branch lengths from the CBFβ phylogeny (*F*_1,17 _= 9.31, *p *= 0.0072, *R*^2^= 0.35).

### *Runx *and *CBFβ *are expressed in overlapping, but not identical, regions of the *Nematostella *head and tentacle ectoderm

**Figure 6:**
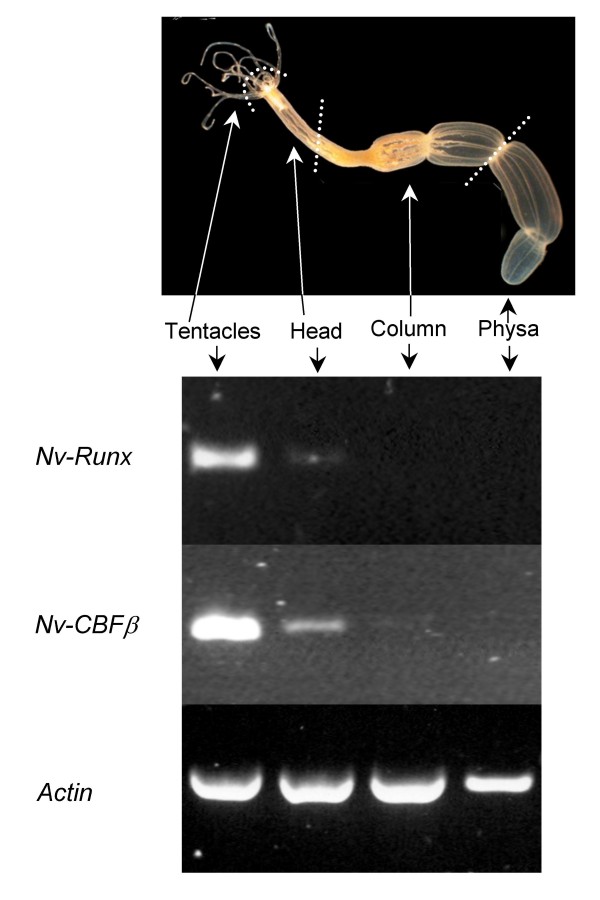
**Expression of *Nv-Runx *and *Nv-CBFβ *in the head region of adult Nematostella anemones**. A single anemone specimen was cut into four parts, as shown in the diagram: tentacles, head/pharyngeal region (including tentacle bases), body column, and physa. Reverse-transcription (RT) PCR was performed on an equal amount of total RNA extracted from each of the four body regions. An equivalent fraction of the amplification reaction was visualized on an agarose gel. Using this assay, *Nv-Runx *expression was detected in the tentacles, and to a lesser extent in the head/pharyngeal region, but not in the column or physa. As a positive control, actin was found to be expressed at high levels in all four body regions.

To determine if expression of *Nv-Runx *and *Nv-CBFβ *is spatially restricted along the primary body axis of the adult polyp we performed reverse-transcription polymerase chain reaction (RT-PCR) on equal amounts of total RNA isolated from four different body regions: (1) tentacles, (2) head region, (3) body column, and (4) physal region. Using two different primer pairs, *Runx *expression was detected in the tentacles and head/pharyngeal region but not the body column or physa (Figure 6). High levels of *CBFβ *expression were observed in the tentacles and head/pharyngeal regions; although a lower level of *CBFβ *expression could also be detected in the body column. No *CBFβ *expression was observed in the physal region.

**Figure 7:**
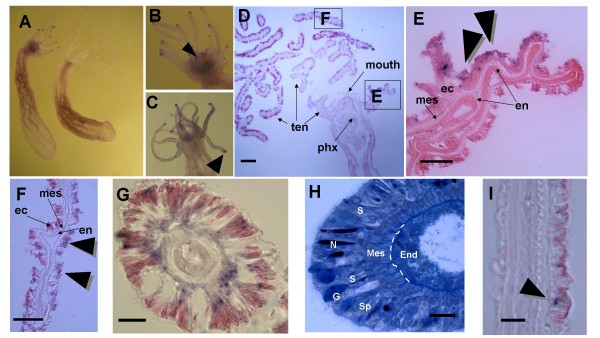
**Expression pattern of *Nv-Runx *in adult *Nematostella***. Digoxigenin-labeled sense-strand and antisense-strand riboprobes corresponding to nucleotides 100–539 of the ORF (exons 1–3, which contain the Runt domain) were used to characterize the spatial expression of Runx in adult *Nematostella*. Similar results were obtained using a longer probe corresponding to the entire *Nv-Runx *transcript (not shown). **A) **Labeled anti-sense probes detect *Nv-Runx *expression in the oral region of the anemones, particularly in the ectoderm of the tentacle tips (animal on left). No specific staining was observed using sense-strand probes (animal on right). **B-C) **While expression was always limited to the tentacle and head region there was some background staining that varied between individual animals. Note that the dark color in the mouth of the animal depicted in panel B (arrowhead) does not represent Runx expression, since it is not detected in sections of this region. The arrowhead in Panel C reveals the strong expression at the tentacle tips. **D-H) **These panels show ectodermal expression of *Nv-Runx *in the tentacles as seen in cryostat sections of anemones after whole mount *in-situ *hybridization. **D) **Low magnification micrograph of a section through the head and tentacles, revealing general architecture as well as the location of the enlarged micrographs in E and F. Bar = 100 μm **E-G) **Expression of *Nv-Runx *in the ectoderm of the tentacles, Bars in E and F = 50 μm, in G = 20 μm, **H) **A thin section of a tentacle from Nematostella, stained with Methylene Blue. Numerous spirocysts (Sp) and several nematocysts (N) can be observed, as can darkly and heterogeneously stained gland cells (G) found towards the apical part of the ectoderm. The elongated cells (S) are probably sensory cells [61]. The mesoglea (Mes) is schematically marked by a dashed line. Bar = 10 μm. **I) **Expression of Nv-Runx in scattered cells in the ectoderm of the body wall (arrowhead). Bar = 20 μm.

We next used whole mount *in-situ *hybridization, followed by cryosectioning, to characterize the spatial expression of *Runx *and *CBFβ*. In agreement with the RT-PCR results, *Nv-Runx *was expressed mainly in the tentacles, with the strongest expression seen in the tentacle tips (Figure 7A). We found that the expression is limited to small foci found exclusively in the ectoderm, often in tight association with nematocytes (Figure 7G). Sporadic *Runx*-expressing cells were also seen in the outer body wall in the pharyngeal region (Figure 7I). The same general pattern of *Runx *expression was observed in ten different anemones using two different anti-sense RNA probes – a 450 nucleotide probe corresponding to exons 1–3 of the gene (containing the Runt domain; Figure 2A) as well as a 1.5 kb probe corresponding to the entire ORF (data not shown). No staining was observed with the complementary negative control sense-strand RNA probes (Figure 7A). While the general pattern of expression was similar in all of the individual anemones, possible differences in the relative level of expression were observed between different animals within the same experiment (compare Figure 7, panels B and C).

**Figure 8:**
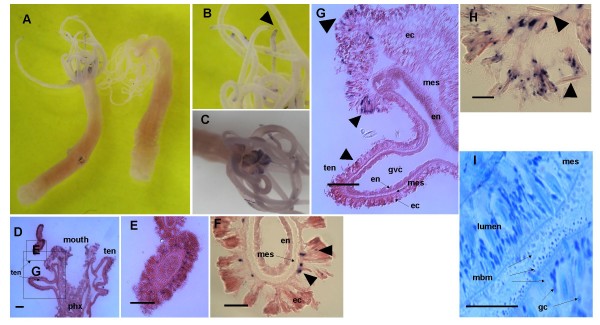
**Expression pattern of *Nv-CBFβ *in adult Nematostella**. A Digoxygenin-labeled riboprobe corresponding to nucleotides 56–434 was used. **A) ***CBFβ *is expressed in the tentacles and mouth region of adult anemones. No staining could be seen when a control probe from the sense strand was used (right animal). **B) **An inset from A, showing strong expression in the tentacle tips (arrow). **C) **Expression of *CBFβ *in the mouth and extended upper pharynx of the same animal as depicted in A and B. Expression levels in the mouth region differed between different animals. **D) **General architecture of the tentacles and mouth region, showing the location of the enlarged micrographs in E and H (panel H is of a different serial section from the same anemone). Ten = tentacle, phx = pharynx. Scale bar = 100 μm. **E, F) **Expression of *CBFβ *at the base of the ectoderm of the tentacles (arrowheads) en = endoderm, ec = ectoderm, mes-mesoglea. Bar in E = 100 μm, in F = 20 μm. **G) **Expression of *CBFβ *in the ectoderm of the mouth (arrows). en = endoderm, ec = ectoderm, mes-mesoglea, gvc = gastrovascular cavity. Bar = 100 μm **H) **Enlargement of a region in the mouth, showing the CBFβ expression in cells close to large microbasic mastigophore nematocysts (arrowheads) Bar = 20 μm. **I) **Thin (2 μm) epoxy cross section through the mouth region stained with Methylene Blue, revealing the secretory gland cells (gc) and abundance of microbasic mastigophore nematocytes (mbm) of different sizes. Bar = 20 μm.

Similar to *Runx*, *CBFβ *is expressed in the tentacle ectoderm, with the strongest staining in the tentacle tips (Figure 8, panels A-C). Also like *Runx*, the *CBFβ*-expressing cells are located primarily in the basal layer of the ectoderm and in close association with large microbasic mastigophore nematocytes, which are a major component of this cell-rich region of the animal (Figure 8. panels F-H). However, in contrast to *Runx*, some of the individual animals assayed also expressed CBFβ in the mouth region (Figure 8, panels D, G, and H).

## Discussion

### Runx and CBFβ Evolution

A primary motivation for performing the phylogenetic analysis was to decipher the relationships among the multiple paralogs that are found in some taxa, and to pinpoint the ancestral lineages in which gene duplications must have occurred in the Runx and/or CBFβ genes. The presence of only a single *Runx *locus in Porifera and Cnidaria, two of the most ancient animal phyla, along with the fact that both the maximum likelihood (ML) and neighbor-joining (NJ) trees can be rooted such that the sponge and cnidarian sequences emerge from the base of the tree, supports the hypothesis that the eumetazoan ancestor possessed only a single *Runx *locus. Subsequently, it appears that this ancestral *Runx *gene underwent independent duplications within arthropods and within vertebrates. While the non-insect arthropods are only known to possess single *Runx *genes, and the Runt1 sequences from spider (*Cupennius*), mite (*Tetranychus*), and crayfish (*Pacifastacus*) cluster together, distinct *Runt*, *Runx*A, and *Lozenge *genes are found in both *Anopholes *and *Drosophila*. *Drosophila *also possesses a fourth Runx paralog, *RuntB*. This requires at least three gene duplications among dipteran insects, with at least two of these gene duplications clearly predating the divergence between fruit fly and mosquito. While only single Runx proteins are known from the non-vertebrate chordates (*Branchiostoma floridae, B. lanceolatum*, and *Ciona*), there are three human paralogs (*Runx 1–3*), which cluster together on the tree. This is not surprising given that two rounds of whole genome duplication are known to have occurred early in vertebrate evolution, prior to the divergence of the ray-finned fishes from the lobe-finned fishes. The genomes of pufferfishes (*Fugu *and *Tetraodon*), which are ray-finned fishes, contain four *Runx *genes [53,54], three of which appear as orthologs of the mammalian *Runx1–3*. The additional *Runx *gene has a relatively short coding region; it has been suggested that it represents an ancestral vertebrate *Runx *that was subsequently lost in the tetrapod lineage [53]. Zebrafish also contain four *Runx *genes, however two of those are clearly *Runx2 *orthologs (*Runx2a *and *Runx2b*) that likely arose from partial or whole genome duplication in the ray-finned fish lineage [55,56]. The two zebrafish Runx2 paralogs have different functions, including the aforementioned role of Runx2b in dorso-ventral axis formation [12]. Thus, the evolution of this gene family displays dynamic changes in gene number as a result of gene duplications but also of possible gene loss.

With respect to CBFβ, the presence of only a single known CBFβ homolog in all taxa except the fruit fly, including other insects (e.g., *Apis *and *Bombyx*) points to a single, relatively recent gene duplication, in the lineage leading to *Drosophila*.

On both the Runx and CBFβ trees, two sea urchin sequences group together with robust bootstrap support (100%), but fail to group with other deuterostome taxa as expected. In the case of *Runx*, the two echinoid sequences (*Strongylocentrotus *and *Heliocidaris*) appear most closely related to an arthropod clade, although with relatively low bootstrap support (65%). In the case of CBFβ, the two echinoid sequences (*Strongylocentrotus *and *Paracentrotus*) fail to group with a fellow echinoderm, the starfish *Asterina*, instead appearing most closely related to a sequence from a snail (*Biomphalaria*). The bootstrap support for this particular grouping is <40%. The low bootstrap support indicates that the echinoids are not exhibiting a strong affinity for protostome sequences so much as they are exhibiting a lack of affinity for the more closely related deuterostome sequences. The simplest explanation for this pattern, which is also supported by echinoid-specific residues in the sequence alignments (Additional files 1, 2) is that these proteins have undergone echinoid-specific sequence changes that obscure their underlying affinity to other deuterostome sequences.

For both proteins, the amino acid substitution rates differ between lineages. In particular, both Runx and CBFβ have diverged rapidly in *Drosphila *and *Caenorhabditis*, resulting in the fruit fly and nematode proteins being less similar to their human homologs than are the cnidarian proteins, despite being more closely related to humans by some 50 million years. *Drosophila *Runx RD's share, on average, ~72% of residues with human Runx proteins (as compared to 81% for *Nematostella*) while *C. elegans and C. briggsae *share, on average, only ~48% residues with human RD. Likewise, the CBFβ proteins from cnidarians share 57–59% of the residues with human CBFβ, respectively, whereas *Drosphila *and *C. elegans *share only ~55% and 22%, respectively. These data are consistent with a number of recent studies that have noted greater genomic similarity between humans and anthozoan cnidarians than between humans and the major protostome model systems [57-59]. It remains to be seen whether the gene duplication events of both *Runx *and *CBFβ *in various lineages are correlated with a diversification of the roles and regulatory networks in these organisms. Such questions may be addressed through functional studies of proteins and computational predictions of regulatory networks.

### Runx and CBFβ binding, co-evolution, and co-expression

The majority of the amino acids involved in the binding of CBFβ to Runx, and the binding of the Runx-CBFβ protein complex to DNA, are highly conserved between human and *Nematostella*. Our comparative protein modeling results show a high degree of stabilizing selection within the Runt domain (RD) and the RD-interacting face of CBFβ. Further, the complete conservation within the metazoa of residues necessary for interaction between the RD and DNA suggests that the RD-CBFβ-DNA complex has been essentially conserved throughout the ~700 million year history of the eumetazoa, in animals whose morphology, ecology, and physiology are as diverse as terrestrial vertebrates, aerial arthropods and marine invertebrates. Interestingly, the CBFβ-binding faces of the sponge proteins are more divergent. Indeed, the *Oscarella *Runx represents the longest branch on the Runx phylogeny. This is interesting given that we failed to recover a CBFβ homolog from this sponge. This higher degree of sequence divergence within the CBFβ-binding face of *O. carmela *Runx combined with the lack of CBFβ expression in developmentally mixed EST pools could indicate CBFβ loss within this demosponge or indicate that this demosponge may deserve a basal position within the metazoan lineage, pre-dating the origin of CBFβ. Targeted sequencing for CBFβ within genomic sequence of *O. carmela *may help resolve this.

Notably, where the *Nematostella *CBFβ protein differs from human, there appear to be compensatory changes in the *Nematostella *RD. The possibility of compensatory substitutions in the RD and CBFβ proteins of sea anemone and human suggests that functional constraints require that directional selection on either protein may promote commensurate divergence in its partner. To test this hypothesis over broad phylogenetic distances, we employed a variation on the protein co-evolution test proposed by [52]. Our results indicate a significant and highly predictive relationship between the branch lengths of Runx and CBFβ phylogenetic trees, providing further support that the evolutionary rates of these two domains are correlated.

However, shared evolutionary histories could result in significant correlations between any pair of proteins [60]. For example, any two proteins will exhibit a correlation if they have evolved in clock-like fashion on the same underlying phylogeny, or if the species from which they are derived differ substantially in their overall rates of molecular evolution. To evaluate the contribution of shared history to the observed correlation, we can compare proteins that do not interact functionally. We observed no correlation between the molecular evolution of Runx and GAPDH, a well-conserved 'house-keeping' gene, and we observed only a weak correlation between CBFβ and GAPDH (Figure 5B). Taken together with the reciprocal amino acid changes that have occurred over deep evolutionary divergences, this strongly suggests that these two proteins have co-evolved from sponges and cnidarians to humans to maintain their ability to co-regulate the transcription of target genes.

### A role for Runx and CBFβ in nerve cells of adult *Nematostella*?

In the adult anemone, *Nv-Runx *is expressed in scattered ectodermal cells, mainly in the tentacles, and predominantly in the tentacle tips. Scattered cells in the body wall ectoderm also express *Runx*. Similarly, *Nv-CBFβ *is expressed most strongly in the ectoderm of the tentacle tips. However, in contrast to *Runx*, *CBFβ *is also expressed in the mouth and upper portion of the pharynx. Thus, *Nv-Runx *and *Nv-CBFβ *are expressed in similar, but possibly not completely overlapping regions of the anemone head and tentacles. Here, as in other systems, including mammals [32] and sea-urchins [11], CBFβ likely functions together with Runx in the sites where they overlap. At other sites, where CBFβ is expressed in the absence of Runx, CBFβ may have other roles, which may involve other partners [11].

The tentacles of sea anemones, where the expression of both *Runx *and *CBF*β is most predominant, are the part of the animal involved in prey capture and manipulation. This region is relatively poor in cell types, with the majority of differentiated cell types present involved either in prey capture (nematocytes, spirocytes) or in its detection (sensory neurons and ganglion cells) (Figure 7H[61]). The nematocytes themselves do not seem to express either *Runx *or *CBFβ*, nor do the gland/mucous cells, which are found exclusively in the apical part of the ectoderm (*Runx *and *CBFβ *expression is seen mainly in the mid and basal parts of the ectoderm). Thus, we speculate that the cells expressing *Runx *and *CBFβ *are probably neuronal cells. Alternatively, they could be precursors of one of the above differentiated cell types – most likely of nematocytes and spirocytes, which are highly enriched in the tentacles, especially towards the distal tips.

In vertebrates, Runx proteins are known to play critical roles in neural differentiation. For example, in mice, *Runx1 *and *Runx3 *influence the development and survival of sensory neurons in several ganglia types such as dorsal root ganglia [24,25,44,45,47]. In mice, *Runx3 *is essential for development of the monosynaptic stretch reflex arc [24,25,62] the simplest and most ancient neuronal circuit. Based on these findings, it was speculated that a *Runx3 *ortholog may play an analogous role in the highly similar anemone nematocysts response circuit [63]. The spatial expression of *Runx *and *CBFβ *in adult *Nematostella *suggests that these proteins might also be involved in neural differentiation.

Taken together, our results suggest a putative role for Runx in the maintenance of specific neurons in the tentacles of adult anemones, in addition to the diversity of other networks and functions in which Runx is also likely involved. In vertebrates, Runx proteins regulate disparate functions at different developmental stages, in each case by interacting with a different regulatory network [8]. It is noteworthy that an EST encoding the *Acropora *ortholog of *CBFβ *was detected in a library from a pre-settlement planula larva stage [58]. This suggests that in corals *Runx *and *CBFβ *are expressed during embryonic and larval development. Future studies involving microarray analysis of gene expression, CHIP-ChIP analysis of *Nv-Runx *target genes, and gene knockdown experiments may help identify the various roles played by Nv-Runx, Nv-CBFβ, and their regulatory networks at different stages in the life history of *Nematostella*.

## Conclusion

In this study, we showed that *Runx *and *CBFβ *are found in the most basal metazoans, cnidarians and sponges, and that in these animals they probably interact and bind DNA in a manner similar to that found in humans. Structural motifs involved in the regulation of the Runx-CBFβ pair, such as the Runx C-terminal Groucho binding motif and the cysteine redox switches, are also conserved, suggesting that other parts of the regulatory network in which Runx and CBFβ are embedded are also evolutionarily conserved. Both Runx and CBFβ are expressed mainly in small cells within the ectoderm of the tentacles, the identity of which still await proof, but may be sensory neurons or their precursors.

## Methods

### Maintenance of Anemones

*Nematostella vectensis *was obtained from the Aquatic Resources Division of the Marine Biological Laboratory at Woods Hole, as well as from the stock maintained at the Finnerty laboratory at Boston University. The anemones were housed in glass dishes of 50% natural seawater/50% distilled water and maintained at 20°C as described previously [64]. The anemones were fed three times a week with freshly hatched nauplii of *Artemia salina*, and the water was changed weekly.

### Identification of novel Runx/CBFβ proteins

Assembled genomes, genomic trace sequences, and/or expressed sequence tags from the following animals were interrogated utilizing a combination of keyword and sequence homology searches: Phylum Protista, *Monosiga brevicollis *and *Proterospongia-like *sp. ATCC50818; Phylum Porifera, *Oscarella carmela *and *Amphimedon queenslandica*; and Phylum Cnidaria, (class Anthozoa) *Nematostella vectensis *and (class Hydrozoa) *Hydra magnipapillata*. The genomes of several additional protozoan species were also searched (Table 1). *Monosiga *and *Proterospongia *ESTs were searched at Choanobase [65,66] using tblastx [67]. A total of 11178 ESTs for *Oscarella carmela *were downloaded from NCBI's Genbank database and searched locally using tblastx, as was a draft assembly of the *Hydra magnipapillata *genome (downloaded from the J. Craig Venter Institute [68]. The *Nematostella *genome was searched using keyword and blastp/tblastx searches of the assembled genome and expressed sequences at the Nematostella vectensis Genomics Database (StellaBase) site [69,70]. Genomic and coding sequences from other taxa were searched at NCBI (Table 1).

### Phylogenetic reconstruction

We utilized the default alignment parameters (protein distance matrix = GONNET 250; gap open penalty = 10.0; end gap penalty = -1.0; gap extension penalty = 0.2; gap distance penalty = 4) in ClustalX [71] to generate multiple sequence alignments for Runx and CBFβ proteins [7,72], Additional files 1, 2). Numerous previously unidentified Runx and CBFβ sequences from diverse taxa were parsed and assembled from the NCBI dbEST database and from whole genome shotgun sequences after tblastn searches (Table 1, [73]. The conserved 128 amino acid Runt domain [7,72] and 135 CBFβ amino acid residues were used for phylogenetic inference (Additional files 1 and 2, respectively).

ProtTest was used to determine which of 80 possible amino acid substitution models is most appropriate for each dataset [74]. Both the tree topology and the model of protein evolution were optimized simultaneously. The Akaike Information Criteria with a modification to control for small sample size (AICc, with alignment length representing sample size; [75]) was used to discriminate among models. Phylogenetic analyses of both Runx and CBFβ revealed rapid lineage divergence in the nematodes (*C. elegans*, *C. brigsae*, and *Diploscapter coronatus*), which suggests that the placement of these taxa could be confounded by long-branch attraction (Additional files 6 and 7, respectively). Because of this, phylogenetic analyses were performed with and without nematode worms for both proteins. The best model identified for Runx was RtREV [76] + γ (ΔAICc = 0). This model was employed in a maximum-likelihood analysis of Runx sequences using the program Phyml [77]. Since RtREV is not available in the Phylip software package (version 3.65), the neighbor-joining analysis was performed using the 2^nd ^best model: JTT [78] + γ (excluding nematodes: ΔAICc = 4.74, including nematodes: ΔAICc = 15.40). For the CBFβ sequences, JTT + γ was used for both maximum likelihood (Phyml) and neighbor-joining (Phylip) analyses for both datasets (*e.g.*, with and without nematodes). The support for individual nodes was assessed using the bootstrap re-sampling method with 1000 replicates [79].

### Distance test for Co-evolution

To determine if the Runt domain and the CBFβ protein are co-evolving, we culled the phylogenetic dataset to include only those taxa for which both Runt and CBFβ sequences are available. While constraining the tree topology such that it reflects the accepted evolutionary relationships among the 11 species for which we identified both proteins [80], we used parsimony (PAUP; Sinauer Associates, Sunderland, Massachusetts) to infer the number of amino acid substitutions that occurred along each branch for both the Runt domain and CBFβ protein. The branch lengths for each tree were plotted against each other. The strength of the correlation between corresponding branch lengths was evaluated using Pearson product moment correlations. Each protein was also independently compared to glyceraldehyde phosphate dehydrogenase (GAPDH) using the same approach. GAPDH is a "housekeeping gene" that serves as a control, because its sequence evolution is not expected to be functionally constrained by the evolution of either Runx or CBFβ. Correlations were considered significant if α < 0.05, as assessed by a linear least squares regression significance test.

### Comparative Modeling of Runx1(RD)-DNA and Runx-CBFβ interfaces in Sponges and Cnidaria

Model structures of the Runt and CBFβ domains of *Nematostella, Hydra, Amphimedon *and *Oscarella *were constructed based on the X-ray structure of the human AML1(RUNX1)-CBFβ-DNA complex (PDB # 1H9D, [81]). The Runt domains from the cnidarians and sponges have a high sequence similarity with the human protein (see Results, below). The *Nematostella *sequence has the same length as the human Runx1 RD sequence whereas the hydra and sponge sequences are 1- or 2-residues longer. The inserts map to a loop that is not involved in either DNA or CBFβ binding. The *Nematostella, Hydra, Amphimedon *and human variants of CBFβ are also highly similar. The major difference between *Nematostella *and human CBFβ is the loss of a five amino acid stretch in the *Nematostella *sequence (Figure 2D). This deletion maps to a loop between Pro70 and Thr80 in the human CBFβ sequence whose structure is not resolved in the experimental structure of RD-CBFβ complex and is not involved in AML1(RUNX1)-CBFβ-DNA complex formation. This loop was not modeled. The *Hydra *protein has one amino acid deleted in this region, whereas the *Amphimedon *CBFβ domain shows a 2-residue insert the same region. The overall high degree of conservation strongly suggests that the overall folds of the Runx(RD) and CBFβ domains studied here are similar to the folds of the corresponding human domains. Therefore, the Cα atoms were constrained to their initial positions during the energy minimization, except for the modified loop in Runx(RD). Models were constructed using the 'Homology' module of InsightII (Accelrys Inc., San Diego CA), soaked in a layer of water and energy minimized using the 'Discover-3' module, while employing the CVFF force field.

The likelihood of the RD-CBFβ interaction in *Nematostella, Hydra *and *A. queenslandica *was estimated by analyzing the conservation of the interface residues. Additional files 3, 4, 5 indicate interface residues which are buried more than 15% at the interface in the human RD-CBFβ-DNA complex (the 15% limit excludes residues at the edge of the interface whose contribution to the stability of the complex may be marginal). The surface area of each residue which is buried at the interface was estimated as the difference between its exposed surface area in the separated domain and in complex. Exposed surface areas were calculated using the Homology module.

### RT-PCR and Cloning of *Nv-Runx *and *Nv-CBFβ *transcripts

RNA was isolated from adult *Nematostella *anemones using Tri-Reagent according to the manufacturer's protocols (Invitrogen, Carlsbad, CA, USA). Reverse-Transcription Polymerase Chain Reaction (RT-PCR) was used to analyze the expression of *Runx *and *CBFβ *transcripts in different body regions of the anemone. Adult anemones were first anesthetized by gradually supplementing the culture medium with 14% w/v MgCl_2_·6H_2_O. Transverse sections were made using a razor blade to divide each animal into four distinct axial regions: (1) tentacles, (2) head/pharyngeal region (containing tentacle bases), (3) body column, and (4) physal region. First-strand cDNAs were synthesized using RevertAid™ H Minus First Strand cDNA Synthesis Kit (Fermentas, Life Sciences, EU). *Runx *and *CBFβ *were amplified from these cDNA pools in a Mastercycler gradient PCR machine (Eppendorf) using Taq DNA polymerase (Fermentas) under the conditions specified by the manufacturer. The oligonucleotides used were: *Nv-Runx *TCTCCGAATTTTGTGTGCAG and TTCTACTGCGGAATCCATCC of exon 1 and exon 3 respectively; *Nv-CBFβ *GAAAACTCGCTCGTGAATCC and AGTCTTTGCTTCGCCTGTTC of exon 1 and exon 5 respectively and actin primers CATGGAATTGTCACAAACTGG and CACACTTCATGATGGAGTTG that are general for cnidarians. PCR products were cloned into pGEM-T-Easy (Promega), and sequenced at the Hebrew University Genome Center.

### *In-situ *hybridization

*In situ *hybridization was performed using the protocol reported by Martinez and co-workers [82] with several modifications. Briefly, medium sized adult *Nematostella *(3 days after last feeding) were transferred into a small dish, allowed to relax for about one hour and anesthetized as described above. After about 10 minutes, the anemones did not respond to tactile stimulation, at which point they were fixed in 4% paraformaldehyde (PFA) in NM for at least four hours at room temperature. The animals were dehydrated through a methanol series, rehydrated into PBST (Phosphate Buffered Saline containing 0.1% Tween-20), and digested for 20 minutes with 0.3 U/ml Proteinase K (Roche). The digestion was terminated by incubation in 4% Glycine in PBST for ten minutes. The animals were then washed twice in PBST, twice in 0.1 M triethanolamine (pH 7.8) twice in 0. 25% (v/v) acetic anhydride in 0.1 M triethanolamine, twice more in PBST, and then refixed for one hour in 4% PFA in PBST. After several more washes in PBST, the anemones were washed once in 50% PBST/50% HS (Hybridization Solution: 50% formamide, 0.1% Tween 20, 5 × SSC, 0.1% CHAPS, 1× Denhardt's solution, 100 μg/ml heparin and 200 μg/ml tRNA), once in 100% HS, and prehybridized in 100% HS for at least two hours at 55°C. Hybridization was performed in HS containing 0.05 ng/μl of the appropriate DIG-labeled probe (produced using the DIG RNA labeling kit, Roche) for 48–72 hours at 55°C. Following hybridization, the animals were taken through a series of seven washes, each lasting 1 hour at 55°C (100% HS/tRNA; 100%, HS/2 × SSC; 75% HS/2 × SSC, 50% HS/2 × SSC; 25% HS/2 × SSC; 2 × SSC/1% CHAPS, 2 × SSC/1% CHAPS). The animals were then transferred to MAB (Maleic Acid Buffer: 0.1 M Maleic Acid, 0.15 M NaCl, pH 7.5), blocked for at least two hours in blocking solution (20% Goat Serum, 1% Bovine Serum Albumin, 0.1% Sodium Azide in MAB) and incubated overnight at 4°C with alkaline phosphatase-conjugated anti-digoxigenin antibodies (Roche) that had been preadsorbed to adult *Nematostella*. Following incubation with the antibody, the animals were washed extensively in MAB over a 24-hour period at room temperature and transferred into NTMT (0.1 M NaCl, 0.05 M MgCl_2 _and 0.1% Tween-20 in 0.1 M Tris-HCl, pH 9.5). Presence of the antibody was detected using the color substrate NBT-BCIP (Roche). The staining reaction was terminated by extensive washing with PBST. Pictures were taken with either an Olympus BX51 microscope equipped with an Optronics camera or an Olympus dissecting microscope (Carl Zeiss, Jena, Germany) equipped with an Olympus DP10 CCD camera (Tokyo, Japan).

### Sectioning of animals after whole mount *in-situ *hybridization

Following whole-mount *in situ *hybridization, animals were washed extensively in PBST, infiltrated with 30% sucrose in PBST as a cryoprotectant, immersed in Tissue-Tek medium and frozen on dry ice or liquid nitrogen. Ten-micron sections were taken using a Leica CM1850 cryostat (Wetzlar, Germany). The sections were counterstained in Eosin and mounted in glycerol without dehydration and tissue clearing, as this can cause loss of the color precipitate. Micrographs were takes with an Olympus BX51 microscope equipped with an Optronics camera. For the histological sections of the mouth region, an anemone was taken after whole mount *in-situ *hybridization with a control probe, refixed in 4% PFA in PBST, dehydrated into isopropanol and embedded in Agar-100 resin. Two-micron sections were taken with an Ultratome-3 (LKB-Prodikter) and stained with Methylene Blue.

## Authors' contributions

JCS, DS, JRF and UG conceived and designed the study. DS, UG, KS and JCS discovered and analyzed the genomic structure of the *Nv-Runx *gene. JCS searched various databases for Runx and CBFβ together with DS and performed the phylogenetic analyses together with AMR and JRF. DS performed the molecular work together with UG and HM contributed to the gene expression studies. ME performed the structural modeling and analyzed the data in collaboration with DS, DL, YG and UG. KS participated in the genomic search for Runx binding sites together with JCS. All authors participated in writing and approval of the final manuscript.

## Supplementary Material

Additional file 1 **Runx alignment used for phylogenetic inference**. Two known redox switches are indicated with red text [34]. The sources of the sequences and the abbreviations for taxa are provided in Additional file 9. Click here for file

Additional file 2 **CBFβ alignment used for phylogenetic inference**. The sources of the sequences and the abbreviations for taxa are provided in Additional file 10. Click here for file

Additional file 3 Conserved Runt domain (RD) residues necessary for DNA binding. Click here for file

Additional file 4 Runx residues which are necessary for RD-CBFβ interaction. Click here for file

Additional file 5 CBFβ residues which are necessary for the RD-CBFβ interaction. Click here for file

Additional file 6 **Runx phylogeny, including nematode lineages**. To test for long-branch attraction, the the phylogenetic relationships among Runx proteins were estimated with and without nematode worms. Neither the topology nor the bootstrap support for individual nodes was significantly affected by exclusion of nematode sequences (compare against Figure 4A). Click here for file

Additional file 7 **CBFβ phylogeny, including nematode lineages**. To test for long-branch attraction, the CBFβ phylogeny was created with and without nematode worms. Neither topology nor bootstrap support was significantly affected by exclusion of nematode sequences (compare against Figure 4B). Click here for file

Additional file 8 **The dimerization interfaces of Nv-Runx and Nv-CBFβ with the position of the interacting amino acid residues noted**. Runx is shown in blue and CBFβ in green. Non conservative replacements are indicated in red. Note that these replacements occur at the edge of the interface. Also, they are reciprocated in the other protein, thus in the RD-CBFβ complex the replacements F153->K121 and H163->C131 in RD are in spatial proximity to the replacements Q67->H67 and F17->M17 in CBFβ, respectively. Click here for file

Additional file 9 Genebank accession numbers and taxon ID's of taxa used in phylogenetic analyses of the Runt domain. Click here for file

Additional file 10 Genebank accession numbers and taxon ID's of taxa used in phylogenetic analyses of CBFβ. Click here for file
