# Supplementary figures and images for: The evolutionary origin of the Runx/CBFbeta transcription factors – Studies of the most basal metazoans
Source: BMC Evol Biol. 2008 Aug 5;8:228. doi: 10.1186/1471-2148-8-228 (PMC2527000; doi:10.1186/1471-2148-8-228)

## Slide 1
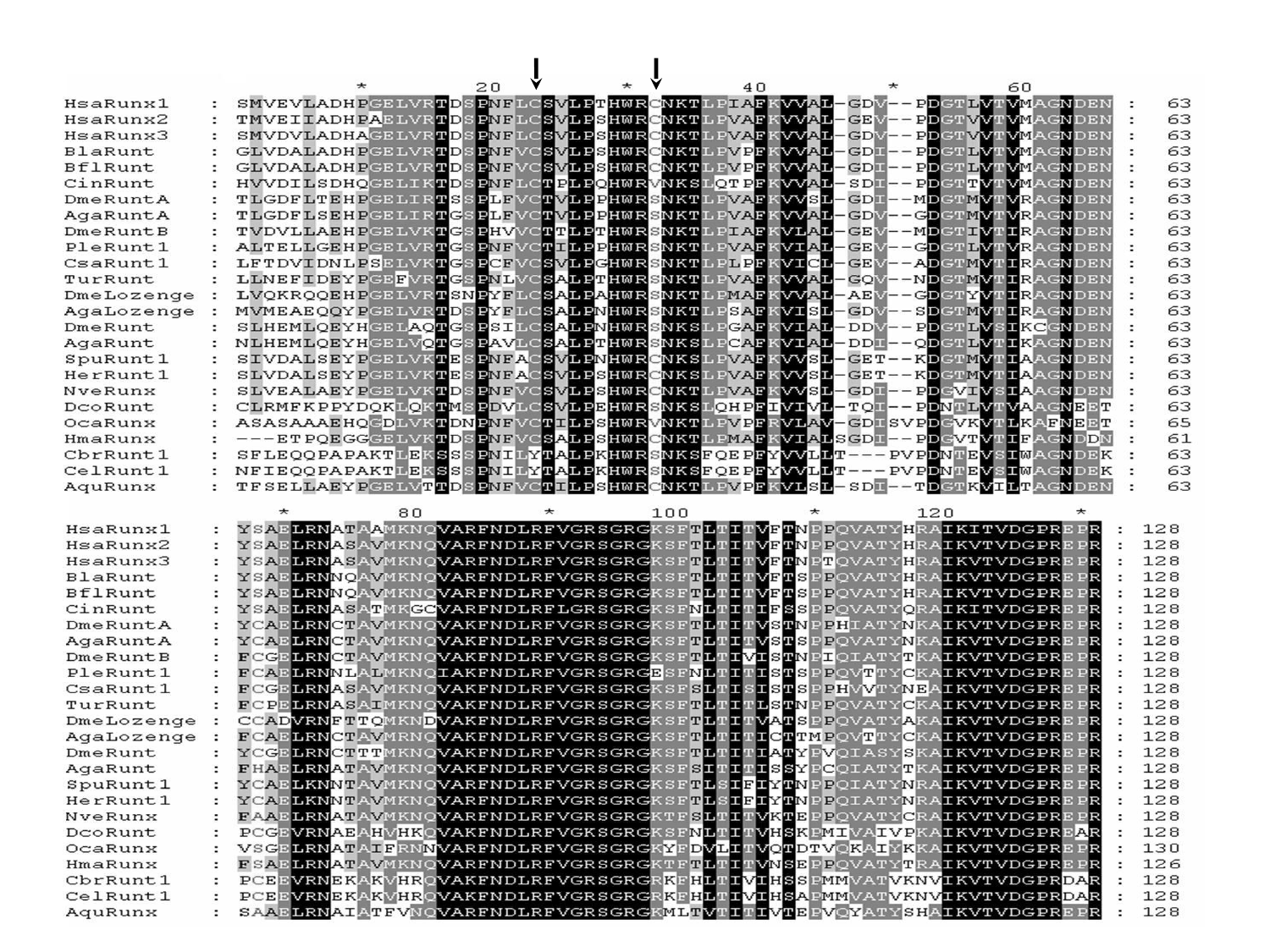

Supplement: Additional file 1 — Runx alignment used for phylogenetic inference. Two known redox switches are indicated with red text [34]. The sources of the sequences and the abbreviations for taxa are provided in Additional file 9. [file 1471-2148-8-228-S1.ppt]

## Slide 1
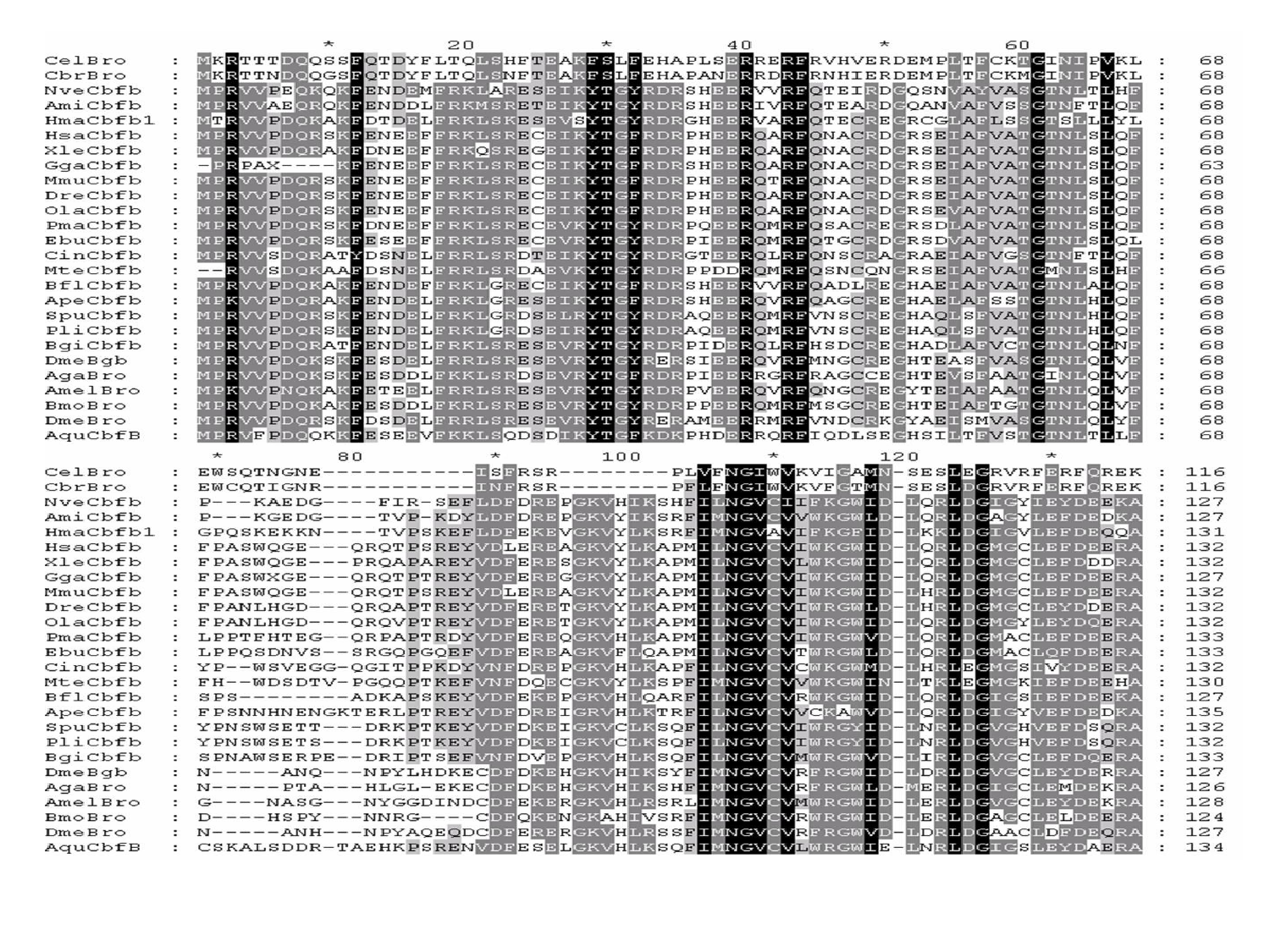

Supplement: Additional file 2 — CBFβ alignment used for phylogenetic inference. The sources of the sequences and the abbreviations for taxa are provided in Additional file 10. [file 1471-2148-8-228-S2.ppt]

## Slide 1
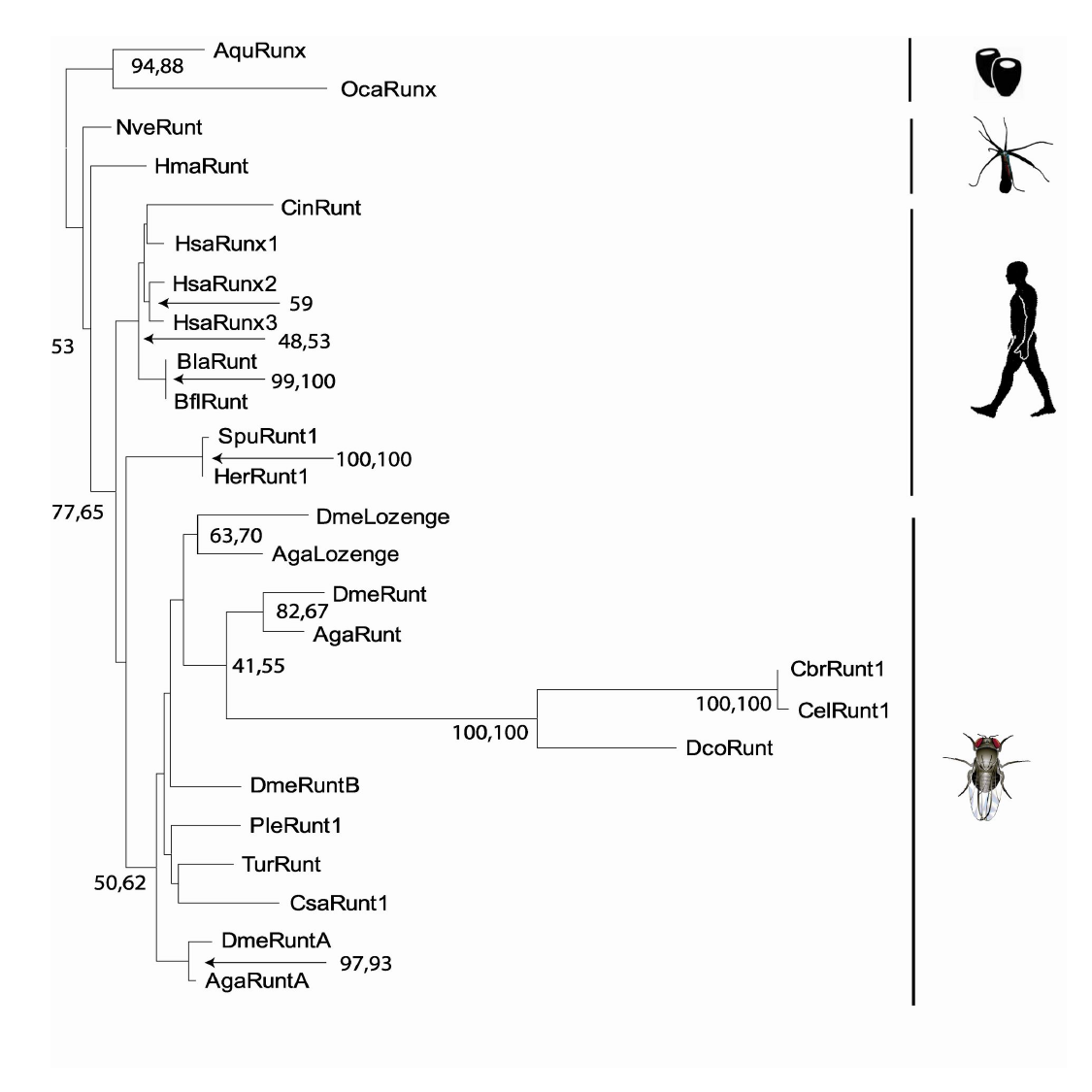

Supplement: Additional file 6 — Runx phylogeny, including nematode lineages. To test for long-branch attraction, the the phylogenetic relationships among Runx proteins were estimated with and without nematode worms. Neither the topology nor the bootstrap support for individual nodes was significantly affected by exclusion of nematode sequences (compare against Figure 4A). [file 1471-2148-8-228-S6.ppt]

## Slide 1
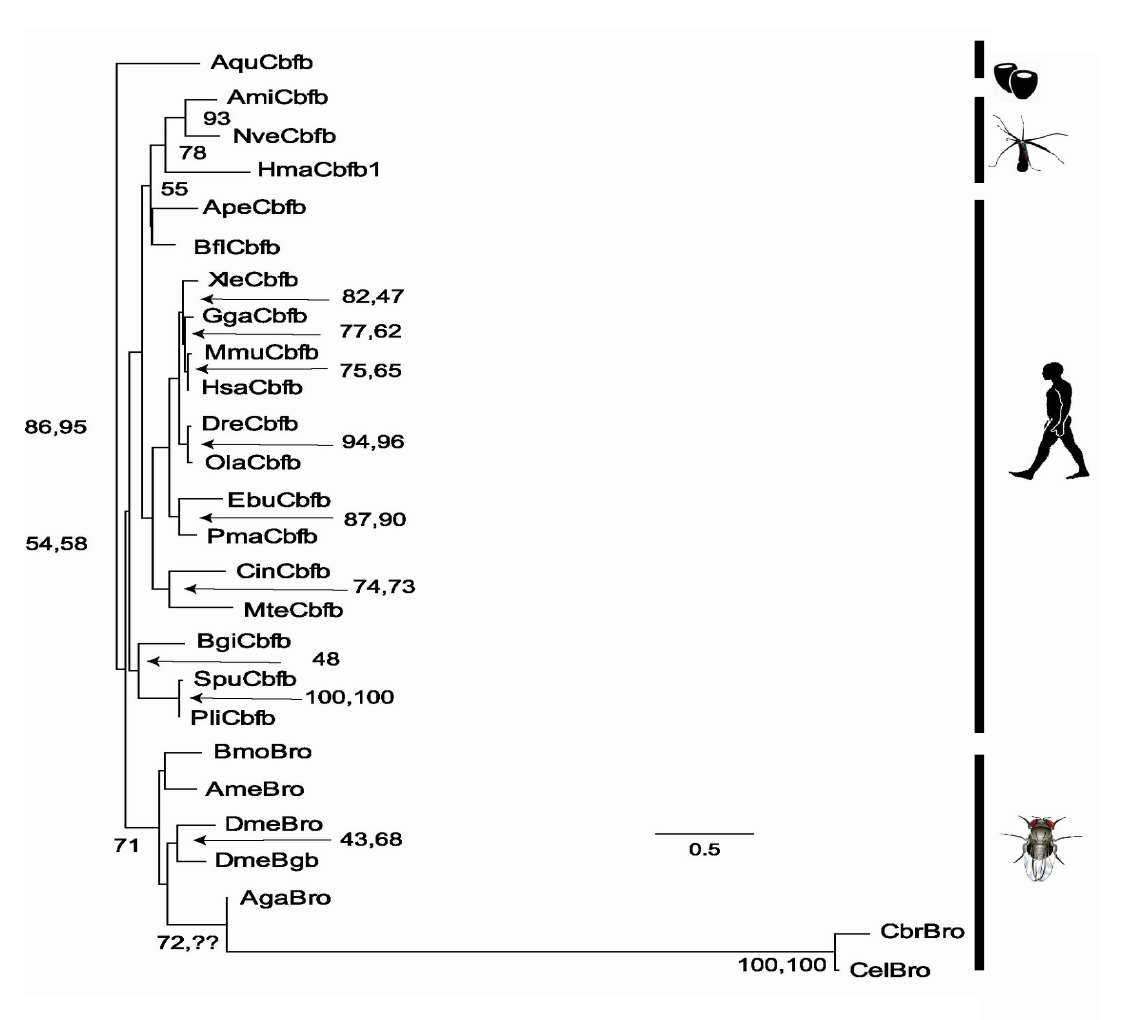

Supplement: Additional file 7 — CBFβ phylogeny, including nematode lineages. To test for long-branch attraction, the CBFβ phylogeny was created with and without nematode worms. Neither topology nor bootstrap support was significantly affected by exclusion of nematode sequences (compare against Figure 4B). [file 1471-2148-8-228-S7.ppt]

## Slide 1
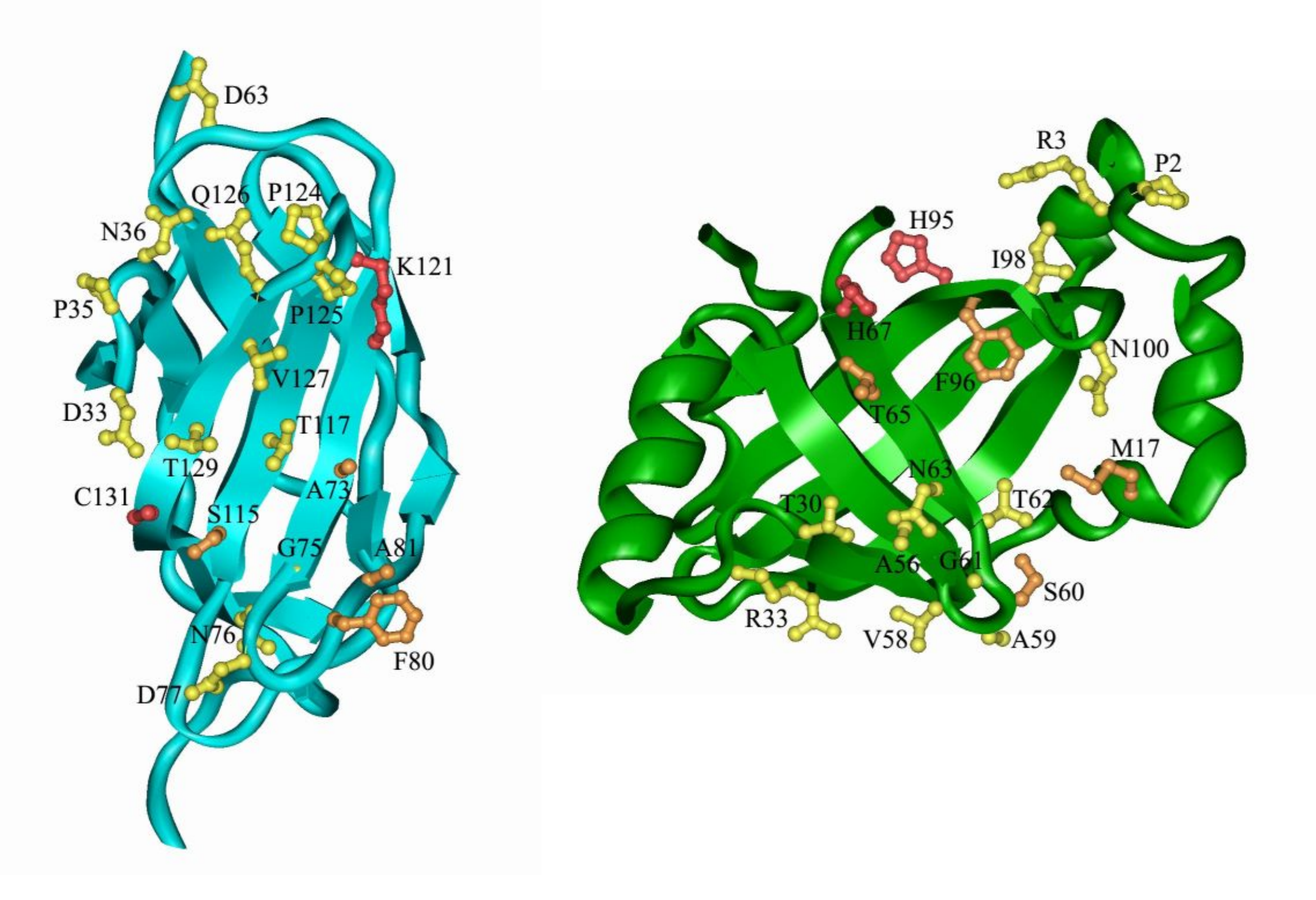

Supplement: Additional file 8 — The dimerization interfaces of Nv-Runx and Nv-CBFβ with the position of the interacting amino acid residues noted. Runx is shown in blue and CBFβ in green. Non conservative replacements are indicated in red. Note that these replacements occur at the edge of the interface. Also, they are reciprocated in the other protein, thus in the RD-CBFβ complex the replacements F153->K121 and H163->C131 in RD are in spatial proximity to the replacements Q67->H67 and F17->M17 in CBFβ, respectively. [file 1471-2148-8-228-S8.ppt]
