# Supplementary material for: The evolutionary origin of the Runx/CBFbeta transcription factors – Studies of the most basal metazoans
Source: BMC Evol Biol. 2008 Aug 5;8:228. doi: 10.1186/1471-2148-8-228 (PMC2527000; doi:10.1186/1471-2148-8-228)
Supplement: Additional file 3 — Conserved Runt domain (RD) residues necessary for DNA binding. [file 1471-2148-8-228-S3.doc]

**Additional file 3**. Conserved Runt domain (RD) residues necessary for DNA binding

| *Homo sapiens* | | Corresponding residue | | | | Inferred | |
| --- | --- | --- | --- | --- | --- | --- | --- |
| Residue | % Buried at Interface | *N. vectensis* | *H. magnipapillata* | *A. queenslandica* | *O. carmella* | Ancestral Metazoan | Ancestral Eumetazoan |
| Arg-80 | 39 | Arg-47 | Arg-57 | Arg-39 | Arg-59 | Arg | Arg |
| Lys-83 | 37 | Lys-50 | Lys-60 | Lys-42 | Lys-62 | Lys | Lys |
| Arg-135 | 15 | Arg-103 | Arg-114 | Arg-95 | Arg-118 | Arg | Arg |
| Arg-139 | 20 | Arg-107 | Arg-118 | Arg-99 | Arg-122 | Arg | Arg |
| Arg-142 | 49 | Arg-110 | Arg-121 | Arg-102 | Arg-125 | Arg | Arg |
| Thr-169 | 27 | Thr-137 | Thr-148 | Thr-129 | Thr-152 | Thr | Thr |
| Val-170 | 33 | Val-138 | Val149 | Val-130 | Val-153 | Val | Val |
| Asp-171 | 38 | Asp-139 | Asp-150 | Asp-131 | Asp-154 | Asp | Asp |
| Arg-174 | 26 | Arg-142 | Arg-153 | Arg-134 | Arg-157 | Arg | Arg |
| Arg-177 | 31 | Arg-145 | Arg-156 | Arg-137 | Arg-160 | Arg | Arg |

Runx residues identified in human as being necessary for Runt domain-DNA interaction are completely conserved in *Nematostella*, *Hydra, A. queenslandica,* and *O. carmella*. The amino acid inferred to have been present at each residue in the ancestral metazoan (*i.e.*, the common animal ancestor) and the ancestral eumetazoan (*i.e.*, the cnidarian-bilaterian ancestor) was identified from parsimony analysis. The % buried at interface relates to the relative importance of this residue in dimerization, as explained under Methods.
