# Supplementary material for: The evolutionary origin of the Runx/CBFbeta transcription factors – Studies of the most basal metazoans
Source: BMC Evol Biol. 2008 Aug 5;8:228. doi: 10.1186/1471-2148-8-228 (PMC2527000; doi:10.1186/1471-2148-8-228)
Supplement: Additional file 4 — Runx residues which are necessary for RD-CBFβ interaction. [file 1471-2148-8-228-S4.doc]

**Additional file 4**. Runx residues which are necessary for RD-CBFβ interaction.

| *Homo sapiens* | | Corresponding residue | | | | Inferred | |
| --- | --- | --- | --- | --- | --- | --- | --- |
| Residue | % Buried at Interface | *N. vectensis* | *H. magnipapillata* | *A. queenslandica* | *O. carmella* | Ancestral Metazoan | Ancestral Eumetazoan |
| Asp-66 | 22 | Asp-33 | Asp-43 | Asp-25 | Asp-45 | Asp | Asp |
| Pro-68 | 61 | Pro-35 | Pro-45 | Pro-27 | Pro-47 | Pro | Pro |
| Asn-69 | 23 | Asn-36 | Asn-46 | Asn-28 | Asn-48 | Asn | Asn |
| Asp-96 | 15 | Asp-63 | Asp-74 | Asp-55 | **^N^**Ser-77 | Asp | Asp |
| Met-106 | 48 | **^N^**Ala-73 | **^N^**Phe-84 | **^N^**Thr-65 | **^N^**Lys-87 | unknown | unknown |
| Gly-108 | 15 | Gly-75 | Gly-86 | Gly-67 | **^N^**Phe-89 | Gly | Gly |
| Asn-109 | 16 | Asn-76 | Asn-87 | Asn-68 | Asn-90 | Asn | Asn |
| Asp-110 | 16 | Asp-77 | Asp-88 | Asp-69 | **^C^**Glu-91 | Asp | Asp |
| Tyr-113 | 70 | **^C^**Phe-80 | **^C^**Phe-91 | **^N^**Ser-72 | **^N^**Val-94 | unknown | unknown |
| Ser-114 | 50 | **^N^**Ala-81 | Ser-92 | **^N^**Ala-73 | Ser-95 | Ser | Ser |
| Thr-147 | 18 | **^C^**Ser-115 | Thr-126 | Thr-107 | **^N^**Asp-130 | Thr | Thr |
| Thr-149 | 32 | Thr-117 | Thr-128 | Thr-109 | **^N^**Leu-132 | Thr | Thr |
| Phe-153 | 25 | **^N^**Lys-121 | **^N^**Asn-132 | **^N^**Val-113 | **^N^**Gln-136 | unknown | unknown |
| Pro-156 | 59 | Pro-124 | Pro-156 | Pro-116 | **^N^**Thr-139 | Pro | Pro |
| Pro-157 | 46 | Pro-125 | Pro-136 | **^C^**Val-117 | **^C^**Val-140 | unknown | Pro |
| Gln-158 | 32 | Gln-126 | Gln-137 | Gln-118 | Gln-141 | Gln | Gln |
| Val-159 | 58 | Val-127 | Val-138 | **^N^**Tyr-119 | **^N^**Lys-142 | Unknown | Val |
| Thr-161 | 28 | Thr-129 | Thr-140 | Thr-121 | **^N^**Ile-144 | Thr | Thr |
| His-163 | 20 | **^N^**Cys-131 | **^N^**Thr-142 | **^N^**Ser-123 | **^N^**Lys-146 | unknown | unknown |

Runx residues identified in human as being necessary for Runt domain-CBFβ-binding are well conserved in *Nematostella* and *Hydra,* moderately conserved in *A. queenslandica,* and poorly conserved in *O. carmella*. Residues preceded with a superscript ‘C’ have conserved physical-chemical properties with the human residue at that position. Those residues preceded with a superscript ‘N’ represent non-conservative substitutions relative to the human variant. The amino acid inferred to have been present at each residue in the ancestral metazoan (*i.e.*, the common animal ancestor) and the ancestral eumetazoan (*i.e.*, the cnidarian-bilaterian ancestor) was identified from parsimony analysis. ‘Unknown’ indicates that the present dataset fails to parsimoniously resolve the residue.
