# Supplementary material for: The evolutionary origin of the Runx/CBFbeta transcription factors – Studies of the most basal metazoans
Source: BMC Evol Biol. 2008 Aug 5;8:228. doi: 10.1186/1471-2148-8-228 (PMC2527000; doi:10.1186/1471-2148-8-228)
Supplement: Additional file 5 — CBFβ residues which are necessary for the RD-CBFβ interaction. [file 1471-2148-8-228-S5.doc]

**Additional file 5.** CBFβ residues which are necessary for the RD-CBFβ interaction.

| *Homo sapiens* | | Corresponding residue | | | Inferred | |
| --- | --- | --- | --- | --- | --- | --- |
| Residue | % Buried at Interface | *N. vectensis* | *H. magnipapillata* | *A. queenslandica* | Ancestral Metazoan | Ancestral Eumetazoan |
| Pro-2 | 38 | Pro-2 | **^N^**Thr-2 | Pro-2 | Pro | Pro |
| Arg-3 | 31 | Arg-3 | Arg-3 | Arg-3 | Arg | Arg |
| Phe-17 | 19 | **^N^**Met-17 | **^N^**Leu-17 | **^N^**Val-17 | unknown | unknown |
| Thr-30 | 18 | Thr-30 | Thr-30 | Thr-30 | Thr | Thr |
| Arg-33 | 29 | Arg-33 | Arg-33 | **^C^**Lys-33 | unknown | Arg |
| Ala-56 | 16 | Ala-56 | Ala-56 | **^N^**Thr-56 | unknown | Ala |
| Val-58 | 18 | Val-58 | **^C^**Leu-58 | Val-58 | Val | Val |
| Ala-59 | 18 | Ala-59 | **^N^**Ser-59 | **^N^**Ser-59 | unknown | unknown |
| Thr-60 | 22 | **^C^**Ser-60 | **^C^**Ser-60 | Thr-60 | unknown | unknown |
| Gly-61 | 55 | Gly-61 | Gly-61 | Gly-61 | Gly | Gly |
| Thr-62 | 20 | Thr-62 | Thr-62 | Thr-62 | Thr | Thr |
| Asn-63 | 64 | Asn-63 | **^N^**Ser-63 | Asn-63 | Asn | Asn |
| Ser-65 | 64 | **^C^**Thr-65 | **^N^**Leu-65 | **^C^**Thr-65 | Thr | Thr |
| Gln-67 | 40 | **^N^**His-67 | **^N^**Tyr-67 | **^N^**Leu-67 | unknown | unknown |
| Pro-100 | 22 | **^N^**His-95 | **^N^**Arg-99 | **^N^**Gln-102 | unknown | unknown |
| Met-101 | 24 | **^N^**Phe-96 | **^N^**Phe-100 | **^N^**Phe-103 | Phe | Phe |
| Ile-102 | 31 | Ile-98 | Ile-102 | Ile-105 | Ile | Ile |
| Asn-104 | 51 | Asn-100 | Asn-104 | Asn-107 | Asn | Asn |

CBFβ residues identified in human as being necessary for Runt domain-CBFβ-binding are well conserved in *Nematostella* and moderately conserved in *Hydra* and *A. queenslandica*. Residues preceded with a superscript ‘C’ have conserved physical-chemical properties with the human residue at that position. Those residues preceded with a superscript ‘N’ represent non-conservative substitutions relative to the human variant. The amino acid inferred to have been present at each residue in the ancestral metazoan (*i.e.*, the common animal ancestor) and the ancestral eumetazoan (*i.e.*, the cnidarian-bilaterian ancestor) was identified from parsimony analysis.‘Unknown’ indicates that the present dataset fails to parsimoniously resolve the residue.
