# Supplementary material for: The evolutionary origin of the Runx/CBFbeta transcription factors – Studies of the most basal metazoans
Source: BMC Evol Biol. 2008 Aug 5;8:228. doi: 10.1186/1471-2148-8-228 (PMC2527000; doi:10.1186/1471-2148-8-228)
Supplement: Additional file 9 — Genebank accession numbers and taxon ID's of taxa used in phylogenetic analyses of the Runt domain. [file 1471-2148-8-228-S9.doc]

**Additional file 9**. Genebank accession numbers and taxon ID’s of taxa used in phylogenetic analyses of the Runt domain.

| **Taxon** | **NCBI Taxon ID** |  | **Sequence ID^§^** | **Genbank**  **Accession Number** | |  |
| --- | --- | --- | --- | --- | --- | --- |
| *Anopheles gambiae* | 7165 | Insect (P#) | Aga RunxA | | AAAB01008921^†^ | |
|  |  |  | Aga Runt | | EAA13524 | |
|  |  |  | Aga Logenze | | AAAB01008959^†^ | |
| *Branchiostoma floridae* (Amphioxus*)* | 7739 | Cephalochordate (D) | Bfl Runt | | AAN08567 | |
| *Branchiostoma lanceolatum* (Amphioxus*)* | 7740 | Cephalochordate (D) | Bla Runt | | AAN08565 | |
| *Caenorhabditis briggsae* | 6238 | Nematode (P) | Cbr rnt-1 | | CAE60460 | |
| *Caenorhabditis elegans* | 6239 | Nematode (P) | Cel rnt-1 | | AAB57715 | |
| *Ciona intestinalis* | 7719 | Ascidian (D) | Cin Runt | | BMC Evol Biol, 2003, 3:4 | |
| *Cupiennius salei* | 6928 | Spider (P) | Csa Runt | | CAB89493 | |
| *Diploscapter coronatus* | 288516 | Nematode (P) | Dco rnt-1 | | Kagoshima et al. 2007 | |
| *Drosophila melanogaster* | 7227 | Insect (P) | Dme RunxA | | Q9VRB1 | |
|  |  |  | Dme RunxB | | Q9VRA8 | |
|  |  |  | Dme Runt | | CAA39817 | |
|  |  |  | Dme Logenze | | AAF35308 | |
| *Heliocidaris erythrogramma* | 7634 | Sea Urchin (D) | Her Runt | | AAC28443 | |
| *Homo sapiens* | 9606 | Mammal (D) | Hsa Runx1 | | NP_001745 | |
|  |  |  | Hsa Runx2 | | CAI13532 | |
|  |  |  | Hsa Runx3 | | NP_001026850 | |
| *Hydra magnipapillata* | 6085 | Cnidarian | Hma Runx | | TBA | |
| *Nematostella vectensis* | 45351 | Cnidarian | Nve Runx | | TBA | |
| *Oscarella carmela* | 386100 | Sponge | Oca Runx | | EC370682^‡^ | |
| *Pacifastacus leniusculus* | 6720 | Crustacean (P) | Ple Runt | | CAD44570 | |
| *Strongylocentrotus purpuratus* | 7668 | Sea urchin (D) | Spu Runt | | AAB03565 | |
| *Tetranychus urticae* | 32264 | Mite (P) | Tur Logenze | | AAN65187 | |

**^§^ ‘***Sequence ID’ refers to Figure 4A, Additional file 1, and Additional file 6*

^†^ *Indicates sequences derived from whole genome shotgun sequence*

^‡^ *Indicates sequence found in NCBI dbEST database*

*# P or D in parenthesis after the organism type represents Protostome or Deuterostome, respectively.*
