# Supplementary material for: The evolutionary origin of the Runx/CBFbeta transcription factors – Studies of the most basal metazoans
Source: BMC Evol Biol. 2008 Aug 5;8:228. doi: 10.1186/1471-2148-8-228 (PMC2527000; doi:10.1186/1471-2148-8-228)
Supplement: Additional file 10 — Genebank accession numbers and taxon ID's of taxa used in phylogenetic analyses of CBFβ. [file 1471-2148-8-228-S10.doc]

**Additional file 10**. Genebank accession numbers and taxon ID’s of taxa used in phylogenetic analyses of CBFβ.

| **Taxon** | **NCBI Taxon ID** |  | **Sequence ID^§^** | **Genbank Accession Number** |
| --- | --- | --- | --- | --- |
| *Acropora millepora* | 45264 | Cnidarian | Ami CBFβ | DY584722.1^‡^ |
| *Anopheles gambiae* | 7165 | Insect (P) | Aga Brother | XP_310806 |
| *Apis mellifera* | 7460 | Insect (P) | Ame Brother | XP_393827 |
| *Asterina pectinifera* | 7594 | Sea Star (D) | Ape CBFβ | DB387666^‡^ |
| *Biomphalaria glabrata* | 6526 | Mollusc (P) | Bgl CBFβ | CK989321^‡^ |
| *Bombyx mori* | 7091 | Insect (P) | Bmo Brother | BAAB01089910^†^ |
| *Branchiostoma floridae* (Amphioxus) | 7739 | Cephalochordate (D) | Bfl CBFβ | BW894738^‡^ |
| *Caenorhabditis briggsae* | 6238 | Nematode (P) | Cbr Bro | CAE56128 |
| *Caenorhabditis elegans* | 6239 | Nematode (P) | Cel Bro | AAK73881 |
| *Ciona intestinalis* | 7719 | Ascidian (D) | Cin CBFβ | BAE06355 |
| *Danio rerio* | 7955 | Fish (D) | Dre CBFβ | NP_954679 |
| *Drosophila melanogaster* | 7227 | Insect (P) | Dme Brother | NP_477066 |
|  |  |  | Dme Big Brother | NP_477065 |
| *Eptatretus burgeri* | 7764 | Hagfish (D) | Ebu CBFβ | BJ652690^‡^ |
| *Gallus gallus* | 9031 | Bird (D) | Gg CBFβ | NP_989901 |
| *Homo sapiens* | 9606 | Human (D) | Hsa CBFβ | NP_074036 |
| *Hydra magnipapillata* | 6085 | Cnidarian | Hma CBFβ | DT605894^‡^ |
| *Molgula tectiformis* | 30286 | Ascidian (D) | Mte CBFβ | CJ368187^‡^ |
| *Mus musculus* | 10090 | Mammal (D) | Mm CBFβ | BAA03426 |
| *Nematostella vectensis* | 45351 | Cnidarian | Nve CBFβ | DV094677.1^‡^ |
| *Oryzias latipes* | 8090 | Fish (D) | Ola CBFβ | BJ732021^‡^ |
| *Paracentrotus lividus* | 7656 | Sea Urchin (D) | Pli CBFβ | AM184919^‡^ |
| *Petromyzon marinus* | 7757 | Lamprey (D) | Pma CBFβ | CO550432^‡^ |
| *Strongylocentrotus purpuratus* | 7668 | Sea Urchin (D) | Spu CBFβ | NM_001040015 |
| *Xenopus laevis* | 8355 | Amphibian (D) | Xla CBFβ | AAH77938 |

**^§^ ‘***Sequence ID’ refers to Figure 4B, Additional file 2, and Additional file 7*

^†^ *Indicates sequences derived from whole genome shotgun sequence*

^‡^ *Indicates sequence found in NCBI dbEST database*
